# Refining the picture: new records to the lichen biota of Italy

**DOI:** 10.3897/mycokeys.82.69027

**Published:** 2021-08-11

**Authors:** Juri Nascimbene, Gabriele Gheza, Josef Hafellner, Helmut Mayrhofer, Lucia Muggia, Walter Obermayer, Göran Thor, Pier Luigi Nimis

**Affiliations:** 1 BIOME Lab, Department of Biological, Geological and Environmental Sciences, Alma Mater Studiorum University of Bologna, Via Irnerio 42, 40126 Bologna, Italy University of Bologna Bologna Italy; 2 Division of Plant Sciences, Institute of Biology, NAWI Graz, University of Graz, Holteigasse 6, 8010 Graz, Austria University of Graz Graz Austria; 3 Department of Ecology, Swedish University of Agricultural Sciences, PO Box 7044, 75007 Uppsala, Sweden University of Trieste Trieste Italy; 4 Department of Life Sciences, University of Trieste, Via L. Giorgieri 10, 34127 Trieste, Italy Swedish University of Agricultural Sciences Uppsala Sweden

**Keywords:** Alps, biodiversity, floristics, herbarium specimens, rarity

## Abstract

Based on the analysis of both historical and recent collections, this paper reports an annotated list of taxa which are new to the lichen biota of Italy or of its administrative regions. Specimens were identified using a dissecting and a compound microscope; routine chemical spot tests and standardized thin-layer chromatography (TLC or HPTLC). The list includes 225 records of 153 taxa. Twenty taxa are new to Italy, the others are new to one or more administrative regions, with 15 second records and 5 third records for Italy. Some of the species belong to recently-described taxa, others are poorly known, sterile or ephemeral lichens which were largely overlooked in Italy. Several species are actually rare, either because of the rarity of their habitats (e.g. old-growth forests), or because in Italy they are at the margins of their bioclimatic distribution. The picture of the lichen biota of Italy has now new pixels, but its grain is still coarse. Further analysis of historical collections, increased efforts in the exploration of some areas, and the taxonomic revision of critical groups are still necessary to provide more complete distributional data for new biogeographic hypotheses, taxonomic and ecological research, and biodiversity conservation.

## Introduction

The lichen biota of Italy is among the best known worldwide thanks to a long-lasting tradition of lichenological studies (Nimis 2018) that has experienced a strong boost after the publication of the first modern checklists (Nimis 1993, 2016), and of the first computer-aided keys (Nimis and Martellos 2020). This is reflected in the steep increase of the number of species known to occur in Italy, more than 550 species having been added between 1993 and 2016 (Nimis 2016). Since 2016, new records (both for the country and for its administrative regions) are constantly being published every year (e.g. Ravera et al. 2020a, b, 2021), which indicates that the exploration of the lichen biota of Italy is still incomplete, and that distributional data of many species are still lacunose (e.g. Martellos et al. 2020). More information is available for the regions of the North, Tuscany and Sardinia (Nimis 2016), all of which are known to host more than 1.000 species each, while most regions of Southern and Central Italy are still insufficiently explored, which may hamper accurate estimations of species rarity (Nimis et al. 2018a), analyses of species richness and composition patterns (Marini et al. 2011), as well as species distribution models (Guttová et al. 2019).

In this work we report the results of the analysis of recent collections and the re-evaluation of herbarium specimens, mainly collected during the last twenty years, in the light of recent taxonomical progress. Twenty taxa are new to Italy, 133 are new to different administrative regions, thus providing a substantial contribution to the knowledge of the lichen biota of Italy.

## Materials and methods

Based on the analysis of herbarium material (BOLO, GZU, M, MOD, TSB, RO, UPS, Herb. Nascimbene and other private herbaria), an annotated list of taxa which are new to the lichen biota of Italy or of its administrative regions, has been prepared. The specimens were identified in the laboratory using a dissecting and a compound microscope. Routine chemical spot tests were performed for most specimens. In some cases (i.e. for sterile crustose lichens) standardized thin-layer chromatography (TLC) was used, following the protocols of Orange (2010), or HPTLC, following Arup et al. (1993).

Nomenclature, as well as synonimization of old records, mainly follow ITALIC 6.0 – The information system on Italian Lichens (Nimis and Martellos 2021), which is continuously updated online. This source was used also for retrieving ecological and distributional information for each taxon.

**Figure 1.**
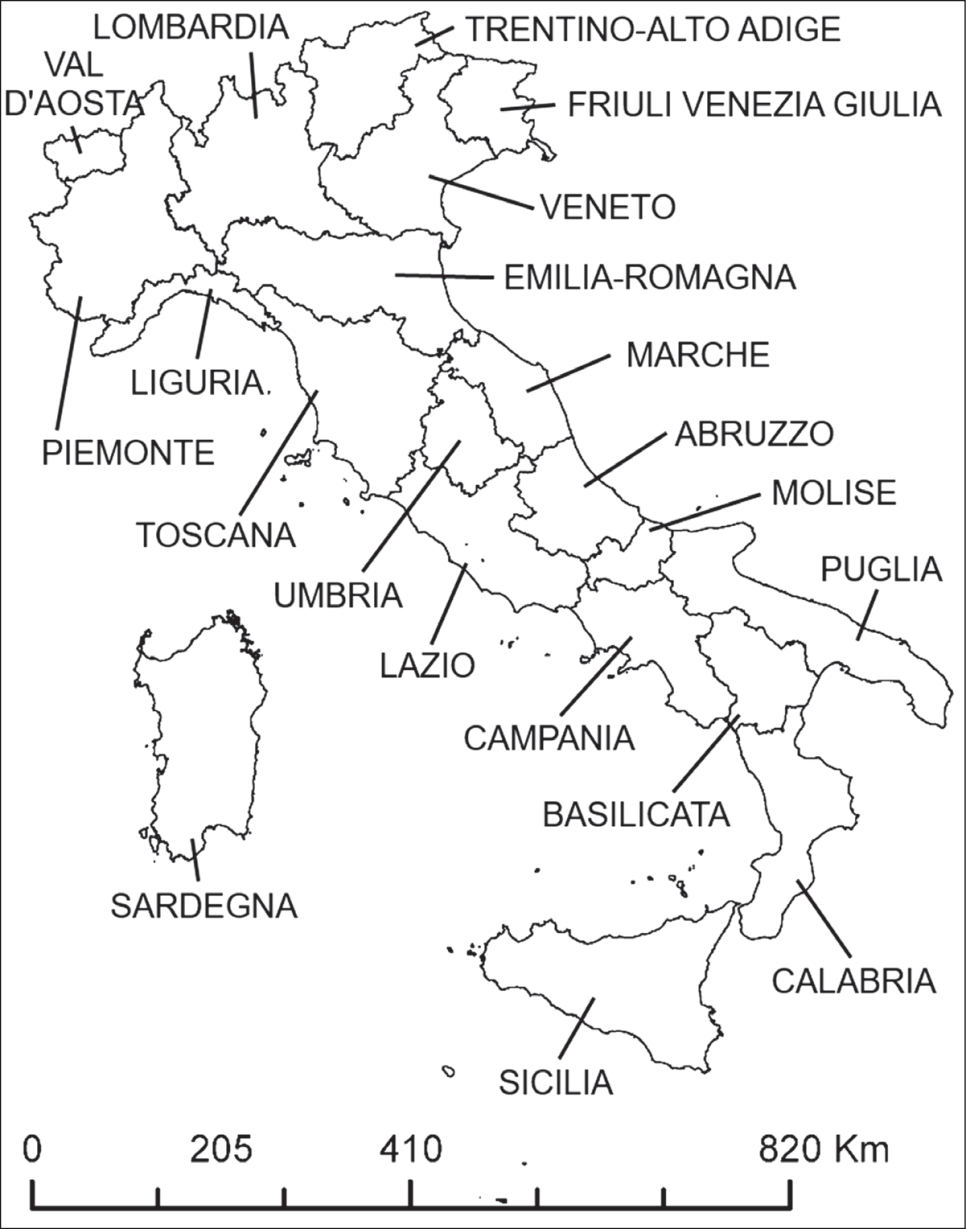
Administrative regions of Italy

Taxa are subdivided in two alphabetically ordered lists: 1) new to Italy, 2) new to administrative regions of Italy (see Fig. 1). For each taxon, the following information is included:

1. administrative region;
2. collection locality, elevation, and substrate. When available, longitude and latitude are reported in DMS. Coordinates available on the original label of the specimens in other formats were converted to this system;
3. collection date, collector(s) name (Leg.), and Herbarium code. Institutional herbaria are abbreviated according to Index Herbariorum (Thiers 2016). The private herbarium of J. Nascimbene is abbreviated as Herb. Na;
4. short note on ecology, distribution and/or taxonomy.

## Results

### Taxa new to Italy

***Agonimiavouauxii* (B. de Lesd.) M. Brand & P. Diederich**

**Abruzzo** • Chieti Prov., Majella National Park, main ridge of the Majella Massif; 42°06'56"N, 14°06'54"E; 2450 m; 12 Jul. 2018; Nascimbene leg.; on soil; Na 5842.

A taxon described from maritime Northern France, where it colonises organic waste like paper, leather, etc.; elsewhere it was reported from soil rich in calcium in open vegetation type.

***Bacidinaadastra* (Sparrius & Aptroot) M. Hauck & V. Wirth**

**Emilia-Romagna** • Parma Prov., Baganzola; 44°51'07"N, 10°18'22"E; 60 m; 03 Mar. 2015; Nascimbene leg.; on *Tilia* sp.; Na 4486, GZU.

This species, similar to *B.arnoldiana*, was described from the Netherlands (Sparrius and Aptroot 2003) and is mainly bound to anthropised environments where it usually grows on acid or neutral, eutrophicated bark, as in the case of our specimen, collected in an urban area.

***Cetreliachicitae* (W.L. Culb.) W.L. Culb. & C.F. Culb.**

**Veneto** • Belluno Prov., Cansiglio Forest, Pian Canaje; 46°06'11"N, 12°24'35"E; 1050 m; 12 Oct. 2020; Nascimbene leg.; on *Fagussylvatica*; Na 6978. – **Friuli Venezia Giulia** • Udine Prov., Lake of Sauris; 46°26'32"N, 12°40'37"E; 1000 m; 03 Sep. 2003; G. Caniglia leg.; on *Fagussylvatica*; Na 5141.

According to Obermayer and Mayrhofer (2007) this is the rarest species of *Cetrelia* in the Eastern Alps.

***Circinariaserenensis* (Cl. Roux & M. Bertrand) A. Nordin**

**Abruzzo** • L’Aquila Prov., Campotosto lake, near Campotosto; 1340 m; 12 Aug. 1996; on limestone; TSB 24336 • L’Aquila Prov., Gran Sasso Massif, Campo Imperatore, 3 km from bifurcation Fonte Velica-C. d. Monte, road to Rifugio Abruzzi; 1500 m; 08 Sep. 1996; Nimis and Tretiach leg.; on limestone; TSB 24460. – **Basilicata** • Potenza Prov., M. Volturino above Marsico Vetere, road to the lift; 1300 m; 11 Apr. 1996; Nimis & Tretiach leg.; on limestone; TSB 22128. – **Campania** • Caserta Prov., road between Sella di Perrone and Campitello Matese, pass between M. Porco and M. La Gallinola; 16 Apr. 2000; Nimis & Tretiach leg.; on limestone; TSB 32108. – **Marche** • Pesaro Prov., Montefeltro, Eremo di Madonna del Faggio above Calvillano di Carpegna; 1300 m; 20 Aug. 1998; Nimis leg.; on limestone; TSB 23373. – **Piemonte** • Cuneo Prov., Cottian Alps, Vallone dell’Arma, on the ridge SE above colle Valcavera; 2470 m; 23 Jul. 2000; Nimis & Tretiach leg.; on limestone; TSB 33966.

A recently-described calcicolous species similar to *C.calcarea*, with optimum in the montane-subalpine belts. The species, which is apparently widespread in the Western Alps (France), might have been filed under *C.calcarea* in the past, and should be looked for throughout the Alps, including the Italian Alps.

***Collemaglebulentum* (Nyl. ex Cromb.) Degel.**

**Trentino-Alto Adige** • Trento Prov., Paneveggio-Pale di San Martino Natural Park, Val Bona; 46°17'35"N, 11°45'36"E; 1740 m; 28 Aug. 2020; Nascimbene leg.; on periodically submerged porphyric rock; Na 6895.

A species found in very humid situations near or above treeline, as in the case of this record, collected on a boulder along a mountain stream.

***Enchyliumcoccophorum* (Tuck.) Otálora, P.M. Jørg. & Wedin**

**Emilia-Romagna** • Bologna Prov., Gessi Bolognesi Natural Park, San Lazzaro di Savena, Dolina della Spipola; 44°26'49"N, 11°22'45"E; 195 m; 07 May 2021; Nascimbene leg.; on terricolous bryophytes above gypsum; Na 7230.

An almost cosmopolitan species of dry areas, found on calciferous soil in dry grasslands, which can be easily mistaken for *E.tenax*. It might be more widespread in Italy, especially in dry Mediterranean areas of the South.

***Fuscopannariaconfusa* (P.M. Jørg.) P.M. Jørg.**

**Trentino-Alto Adige** • Trento Prov., Paneveggio-Pale di San Martino Natural Park, Val Ceremana; 46°17'19"N, 11°43'35"E; 1760 m; 10 Sep. 2012; Nascimbene leg.; on *Salixcaprea*; Na 2819.

A rare European species with a boreal to temperate-high montane distribution, found on branches of various trees and shrubs near the ground, but also on rocks in very humid places, like in the spray zone of waterfalls. Our specimen was found on bark of *Salixcaprea* in a very humid old-growth spruce forest, together with other suboceanic cyanolichens (e.g. *Pannariaconoplea*, *Peltigeracollina*, *Nephromaparile*).

***Gyalideopsishelvetica* van den Boom & Vězda**

**Trentino-Alto Adige** • Trento Prov., Stelvio National Park, Val de la Mare along the path from the parking place at the hydro-electric power station just below Malga Mare; 46°24'58"N, 10°40'53"E; 1990 m; 27 Jul. 2006; Thor leg.; on a *Larix* log; UPS L-166763.

When sterile, as in the case of our collection, the species is recognised by the thallus which consists of a thin film interrupted by patches of concave goniocystangia (van den Boom and Vězda 2000). Described from Switzerland, but widely distributed in Europe, North America and Asia.

***Lecanoraleptacinella* Nyl.**

**Trentino-Alto Adige** • Trento Prov., Paneveggio-Pale di San Martino Natural Park, Passo Rolle, northern slopes of Mt. Tognazza; 46°17'20"N, 11°47'05"E; 2100 m; 01 Sep. 2002; Hafellner leg.; on bryophytes (moribund *Polytrichumsexangulare*); GZU – Ha 63986.

This inconspicuous species settles in a narrow ecological niche, growing on the upper parts of moribund *Polytrichum*-gametophytes, in the Alps only at high altitudes. For a detailed treatment see Obermayer and Poelt (1994).

***Myriosporascabrida* (Hedl. ex H. Magn.) K. Knudsen & Arcadia**

**Toscana** • Livorno Prov., Arcipelago Toscano National Park, Elba Island, M. Capanne; 42°46'16"N, 10°10'02"E; 1000 m; 30 Aug. 1982; Mayrhofer leg.; on granite; GZU – Ma 3923.

A species with an epilithic thallus consisting of pale grey to pale brown areolate to subsquamulose areoles, and 1–2 mm wide apothecia with brown, somewhat raised discs; usually on acidic schistose rocks .

***Protoblasteniaszaferi* J. Nowak**

**Friuli Venezia Giulia** • Udine Prov., Carnic Alps, Monte Tinisa, Le Forcelle; 46°25'10"N, 12°42'10"E; 1900 m; 19 Aug. 1994; Hafellner leg.; on calcareous rock; GZU – Ha 78786.

The species is recognised by its flat, hardly protruding, orange apothecia dispersed on a whitish to cream-coloured semi-endolithic thallus. It grows on steep, shaded faces of limestone. For a detailed treatment see Hafellner (2006).

***Rhizocarponintermediellum* Räsänen**

**Lombardia** • Brescia Prov., Adamello Natural Park, Passo Gallinera; 46°10'49"N, 10°24'49"E; 2320 m; 16 Jun. 2004; Nascimbene leg.; on siliceous rock; Na 5104.

An arctic to nemoral-alpine species morphologically resembling *R.norvegicum*, but ascospores submuriform, larger (to 20 µm long), with 1–4 transverse septa and 1 incomplete longitudinal septum. It colonises basic siliceous rocks and schists with low content of calcium in exposed situations, and starts the life-cycle on various crustose lichens. Most records from the Alps are in the central-eastern part (Nimis et al. 2018a), but the species is likely to have been overlooked in some regions.

***Rhizocarponpostumum* (Nyl.) Arnold**

**Friuli Venezia Giulia** • Udine Prov., Carnic Alps, road from Timau to Passo di M. Croce Carnico, 1100 m; 28 Oct. 1982; P.L. Nimis leg.; M. Tretiach det.; on Werfen sandstone; TSB 2857.

A species recalling *Rh.distinctum* in the small-sized apothecia, but medulla not amyloid and with stictic acid, apothecia flat, smooth, less than 0.5 mm across, ascospores small (mostly less than 25 µm long), submuriform; on siliceous rocks, often close to streams and waterfalls; widespread in Europe, but rather rare or not always distinguished, with scattered records from the Alps.

***Rinodinainterpolata* (Stirt.) Sheard**

**Calabria** • Reggio Calabria Prov., Aspromonte, Pietra Impiccata; 1750 m; 12 Jul. 1988; Josef Poelt leg.; M. Giralt and H. Mayrhofer rev.; on north–facing, deep overhangs of siliceous rock; GZU.

This species is characterized by a pale brown thallus, discrete, sessile apothecia and narrowly ellipsoid *Physcia*-type ascospores grading into the *Physconia*-type at maturity, with a poorly developed torus. It grows on hard siliceous rocks, usually on sheltered and shaded, vertical or overhanging cliffs and boulders. Described from Scotland where it is uncommon (Mayrhofer and Poelt 1979; Giavarini et al. 2009) and also known from scattered localities in middle and southern Scandinavia and Iceland (Mayrhofer and Moberg 2002). Very rare in Western and Central Europe (Giralt et al. 1997; Wirth et al. 2013), being known from mid- to rather high altitudes in Portugal (Giralt 2001).

**SolorinabisporaNyl.var.subspongiosa (Zschacke) Frey**

**Veneto** • Belluno Prov., Dolomiti Bellunesi National Park, Vette Feltrine, near Passo delle Vette Grandi; 46°05'25"N, 11°50'34"E; 2030 m; 10 Apr. 2021; Nascimbene leg.; on soil; Na 7160.

This is probably a morph with a dominant cyanobacterial photobiont in the *S.bispora*-group, growing on basic to subneutral soil at high elevations. A detailed DNA analysis of this group is being carried out by colleagues from Graz.

***Staurothelesapaudica* Asta, Clauzade & Cl. Roux**

**Veneto** • Belluno Prov., Dolomiti d’Ampezzo Natural Park, Tofana di Rozes; 46°32'29"N, 12°03'01"E; 2870 m; 05 Aug. 2020; Nascimbene leg.; on dolomite (dolomia principale) with water seepage; Na 6886, TSB42610.

This species, described from the Western Alps (France), is found on periodically moist calcareous rocks, e.g. along streams at high elevations, or in shaded-humid situations. Our specimen was collected on dolomite washed by melting water in the nival belt.

***Thelidiumschibleri* Zschacke**

**Trentino-Alto Adige** • Trento Prov., Paneveggio-Pale di San Martino Natural Park, above Rolle pass, near Baita Cervino; 46°17'51"N, 11°55'15"E; 2090 m; 14 Sep. 2020; Nascimbene leg.; on sedimentary, calcareous-arenaceous rocks; Na 7131.

A critical taxon, hitherto known only from the type locality in Switzerland where it was collected on calcareous rocks in upland areas. Similar to other *Thelidium*-species, this taxon would require further research to clarify its taxonomic position. Our specimen was collected in a very humid wall of the early Triassic Werfen-formation composed of carbonatic, terrigenous and mixed, varicolored deposits that are sometimes dolomitized. The ecological conditions of the site are similar to those of the type locality, where the species was found together with *Polyblastiacupularis*, as in our case.

***Thelidiumsubabsconditum* Eitner**

**Trentino-Alto Adige** • Trento Prov., Paneveggio-Pale di San Martino Natural Park, Castellazzo; 46°18'29"N, 11°47'51"E and 46°18'26"N, 11°47'43"E; 2198 m; 14 Sep. 2020; Nascimbene leg.; on calcareous rocks; Na 7198.

This species is widespread in the Alps (Nimis et al. 2018a) on inclined surfaces of compact calciferous rocks in rather shaded, non-eutrophicated situations near or above treeline. Since it was not always distinguished from similar species, it has an apparently incomplete distribution in the Alps.

***Toensbergialeucococca* (R. Sant.) Bendiksby & Timdal**

**Trentino-Alto Adige** • Trento Prov., Paneveggio-Pale di San Martino Natural Park, Val Ceremana; 46°17'16"N, 11°43'15"E; 1550 m; 10 Sep. 2012; Nascimbene leg.; on *Piceaabies* in an old-growth forest; Na 3141. – **Friuli Venezia Giulia** • Udine Prov., Carnic Alps, Sauris Lake, Bosco della Stua; 46°26'35"N, 12°42'50"E; 1100 m; 16 Aug. 1994; Hafellner leg.; on *Alnusincana*; GZU – Ha 84342.

This is an obligately sterile species with a thallus consisting of scattered, whitish, adnate areoles and usually marginal soralia, containing alectorialic acid and therefore turning reddish with age. It is widespread in the Holarctic region from the boreal to the nemoral-montane zone, including the Alps (most records being from the eastern part), but was still largely overlooked in Italy, where it may be more widespread, at least in the Alps.

***Usneaflavocardia* Räsänen**

**Sardegna** • Oristano Prov., Seneghe, Montiferru, Nuraghe Ruju; 40°06'49"N, 08°34'55"E; 780 m; 10 Mar. 2014; Nascimbene leg.; on *Quercusilex*; Na 4403.

A Mediterranean-Atlantic species growing on deciduous trees (e.g. *Fagus*, *Quercus*, *Salix* and *Sorbus*). In Europe it is known from France, Great Britain, Greece, Ireland, Netherlands, Portugal, Spain (Randlane et al. 2009), and Germany (Otte 2011). Our specimen was collected in a montane, old-growth, unmanaged forest hosting several suboceanic and oceanic lichens such as *Lobariapulmonaria*, *Ricasoliaamplissima*, and *R.virens*.

### Taxa new to administrative regions of Italy

***Acarosporafreyi* H. Magn.**

**Basilicata** • Potenza Prov., Mt. Volturino above Marsico Vetere; 1500 m; 11 Apr. 1996; Nimis and Tretiach leg.; K. Knudsen rev.; on siliceous rocks, parasitic of *Aspicilia* sp.; TSB 22197. – **Calabria** • Cosenza Prov., Sila Greca, loc. Finaita; 1000 m; 15 Jul. 1988; Nimis, Tretiach and M. Castello leg.; K. Knudsen rev.; on siliceous rock, parasitic on *Aspicilia* sp.; TSB 10995.

Probably overlooked and more widespread, both in the Alps and in the Apennines, with optimum near and above treeline, this lichen starts the life-cycle on species of *Aspicilia*, especially *A.candida* and *A.polychroma*, on calciferous rocks which are at least partly decalcified on the surface.

***Acarosporagallica* H. Magn.**

**Trentino-Alto Adige** • Bolzano/Bozen Prov., Venosta valley, near Silandro/Schlanders; 46°37'50"N, 10°46'46"E; 750–800 m; 12 Sep. 1970; Josef Poelt leg.; on south-facing slopes, on rock; GZU.

A probably holarctic species of base-rich, weakly calciferous siliceous substrata, such as calcareous sandstone, brick, and roofing tiles, usually at relatively low elevations; much overlooked or confused with other species and certainly more widespread in Italy.

***Acarosporairregularis* H. Magn.**

**Basilicata** • Potenza Prov., Melfi, below the castle; 530 m; 14 Apr. 1997; Nimis and Tretiach leg.; K. Knudsen rev.; on basaltic rocks; TSB 29919 (as *Acarosporaoligospora*).

A species known from Central Europe (Austria, Czech Republic, Hungary and Slovakia), as well as Greece and Sardinia, which was often confused with *A.nitrophila* and related species.

***Acarosporalaqueata* Stizenb.**

**Veneto** • Belluno Prov., Dolomiti Bellunesi National Park, Vette Feltrine, Colle Cesta; 46°05'24"N, 11°50'37"E; 2010 m; 13 Jun. 2020; Nascimbene leg.; on selciferous calcareous rocks (Formazione di Fonzaso); Na 7245. – **Sicilia** • Palermo Prov., Madonie, Piano Battaglia; 37°52'47"N, 14°01'31"E; 1600 m; 31 May 1988; Josef Poelt leg.; on calcareous rocks; GZU.

On hard calcareous rocks, both on vertical faces and at the top of birds’ perching sites in dry-continental areas, below the subalpine belt.

***Acarosporasimilis* H. Magn.**

**Veneto** • Belluno Prov., Dolomiti Bellunesi National Park, Erera-Brendol; 46°09'44"N, 11°58'57"E; 1710 m, 18 Oct. 2020; Nascimbene leg.; on a wooden fence near a hut; Na 7011. **Second record for Italy.**

The only previous record of this lignicolous species from Italy is that of a specimen collected on a horizontal wood fence in a vineyard near Merano, at c. 500 m (Nimis 2016). Our sample was collected on a similar substrate but at higher elevation, indicating that in Italy this species could span a wide altitudinal range.

***Acoliummarcianum* (B. de Lesd.) M. Prieto & Wedin**

**Emilia-Romagna** • Bologna Prov., Camugnano, Alto Reno Terme; 44°08'00"N, 11°05'44"E and 44°08'07"N, 10°53'56"E; 870–890 m; 2018; S. Gambini leg.; on old *Castaneasativa* in traditional orchards; Na 6817.

A rare lichen usually growing on pertusarioid silicicolous species, mainly Tyrrhenian in Italy. Our specimen was collected in old-growth *Castanea*-stand near Bologna (Pezzi et al. 2020).

***Agonimiagelatinosa* (Ach.) M. Brand & Diederich**

**Veneto** • Belluno Prov., Dolomiti Bellunesi National Park, Vette Feltrine, near Passo delle Vette Grandi; 46°05'25"N, 11°50'34"E; 2030 m; 10 Apr. 2021; Nascimbene leg.; on terricolous bryophytes; Na 7158. • Belluno Prov., Dolomiti Bellunesi National Park, Vette Feltrine, Cordin delle Vette; 46°05'20"N, 11°51'09"E; 1950 m; 13 Jun. 2021; Nascimbene leg.; on terricolous bryophytes; Na 7224.

A species growing on plant debris and mosses in calcareous dry grasslands, with optimum above treeline.

***Agonimiaopuntiella* (Buschardt & Poelt) Vězda**

**Veneto** • Belluno Prov., Feltre, Vincheto di Celarda Natural Reserve; 46°36'39"N, 11°04'29"E; 310 m, Aug. 2011; Nascimbene leg.; among mosses on isolated *Ulmus* sp.; Na 2558. • Belluno Prov., Feltre, Vincheto di Celarda Natural Reserve; 46°00'50"N, 11°58'41"E; 310 m, Aug. 2011; Nascimbene leg.; among mosses on isolated *Populus* sp.; Na 2560.• Belluno Prov., Feltre, Rocchetta di San Vittore; 46°00'11"N, 11°56'43"E; 400 m; 16 May 2021; Nascimbene leg.; on plant debris and mosses; Na 7203.

A mild-temperate species that in Italy occurs along a wide altitudinal gradient, from the Mediterranean to the montane belt (Nimis 2016) both on terricolous mosses and plant debris over calcareous substrata and amongst mosses on basal parts of old trees.

***Alyxoriaochrocincta* (Werner) Ertz**

**Abruzzo** • Chieti Prov., Abetina di Rosello Natural Reserve; 41°52'36"N, 14°21'23"E; 1000 m; Jul. 2009; Nascimbene leg.; on wood; Na 2416.

A Mediterranean species occurring in shaded and humid situations, as in the case of our sample.

***Anaptychiabryorum* Poelt**

**Piemonte** • Cuneo Prov., Cottian Alps, W ridge of Mt. Nebin, ca. 1 km E of Colle di Sempeyre; 2380 m; Summer 2000; Nimis and Tretiach leg.; TSB 32957.

An arctic-alpine to boreal-montane, probably circumpolar species found amongst mosses and moribund plants on base-rich siliceous substrata in the alpine and subalpine belts.

***Anematumidulum* Henssen ex P.M. Jørg., M. Schultz & Guttová**

**Veneto** • Belluno Prov., Dolomiti Bellunesi National Park, Erera-Brendol, climbing area near San Vito-Primolano; 45°57'06"N, 11°43'28"E; 370 m; 26/10/2019; Nascimbene leg.; compact calcareous rock with water seepage; Na 6816. • Belluno Prov., Dolomiti Bellunesi National Park, Erera-Brendol, climbing area near San Vito-Primolano; 46°09'39"N, 11°58'35"E; 1700 m; 18/10/2020; Nascimbene leg.; compact calcareous rock with water seepage; Na 7031.

Apparently widespread in Central Europe, but poorly collected elsewhere, growing on steeply inclined, sunny surfaces of calcareous or basic siliceous rocks with periodical water seepage after rain.

***Arthoniacalcicola* Nyl.**

**Veneto** • Belluno Prov., Dolomiti Bellunesi National Park, Vette Feltrine, between Piadoch and Cima Dodici, near Busa delle Vette; 46°06'28"N, 11°50'23"E; 2150 m; 19 Jun. 2021; Nascimbene leg.; on marly limestone (Rosso Ammonitico Superiore); Na 7242.

An early coloniser on exposed calcareous rocks below the subalpine belt; overlooked and probably more common, especially in the EU-Mediterranean belt. It also occurs in warm-dry Alpine valleys.

***Arthoniavinosa* Leight.**

**Abruzzo** • Chieti Prov., Abetina di Rosello Natural Reserve; 41°52'55"N, 08°21'24"E; 950–1000 m; Jul. 2009; Nascimbene leg.; on *Abiesalba*; Na 2538. • Ibidem; 23 Oct. 2015; Nascimbene leg.; Na 4717, 4720.

A mild-temperate lichen found near the base of old trees, mostly on rough bark, especially of *Quercus* sp., more rarely on wood, in very humid and closed-canopied forests. Included in the Italian red list of epiphytic lichens as “Near-threatened” (Nascimbene et al. 2013).

***Aspiciliacandida* (Anzi) Hue**

**Veneto** • Belluno Prov., Dolomiti Bellunesi National Park, Vette Feltrine, Passo delle Vette Grandi; 46°05'24"N, 11°50'37"E; 2010 m; 27 Dec. 2003; Nascimbene leg.; on a selciferous carbonatic rock; Na 84. • Ibidem; 26 Jul. 2004; Nascimbene leg.; Na 85. • Belluno Prov., Dolomiti Bellunesi National Park, Vette Feltrine, Cima Dodici; 46°06'30"N, 11°50'30"E; 2200 m; 13 Jun. 2020; Nascimbene leg.; on a selciferous carbonatic rock; Na 6834.

On weakly calciferous rocks, mostly near or above treeline. Our samples were collected on the selciferous carbonatic rock of the late Jurassic formation called “Formazione di Fonzaso”.

***Aspiciliagrisea* Arnold**

**Lombardia** • Brescia Prov., Adamello Natural Park, Edolo, Passo Gallinera; 46°10'49"N, 10°24'49"E; 2320 m; 16 Jun. 2004; Nascimbene leg.; on siliceous rocks; Na 5393. • Brescia Prov., Adamello Natural Park, Presanella-group, Passo del Tonale, S above the pass towards Passo del Paradiso; 46°15'10"N, 10°34'45"E; 1950 m; 24 Jul. 2006; Hafellner and Muggia leg.; gentle N-exposed slope, on inclined faces of large granitic boulders; GZU – Ha 85838. **Second record for Italy.**

A chemically variable species found on siliceous rocks, sometimes also on pebbles, widespread in the Alps (Nimis et al. 2018a), but very much overlooked in Italy.

***Aspiciliamashiginensis* (Zahlbr.) Oxner**

**Lombardia** • Brescia Prov., Adamello Natural Park, Edolo, Passo Gallinera; 46°10'49"N, 10°24'49"E; 2320 m; 16 Jun. 2004; Nascimbene leg.; on siliceous rocks; Na 5394. • Ibidem; 27 Jul. 2006; Hafellner leg.; GZU – Ha 85843, Ha 85844.

A species with grey thalli showing somewhat effigurate margins, the central parts covered in short, thick, hollow papillate structures gradually breaking down into flattened propagules (Poelt 1994). It is restricted to subvertical to overhanging faces of siliceous cliffs from treeline to the nival belt. At the investigated site several thalli have been found, colonised by parasitic *Lecideatessellata*.

***Bacidiaarceutina* (Ach.) Rehm & Arnold**

**Veneto** • Belluno Prov., Feltre, Vincheto di Celarda Natural Reserve; 46°00'49"N, 11°58'37"E; 310 m; Aug. 2011; on *Populus* sp. in a riparian forest; Nascimbene leg.; on *Populus* sp. in a riparian forest; Na 2555. – **Sardegna** • Nuoro Prov., Barbagia Seulo, State Forest of Monte Arbo, S. Girolamo Valley; 900–1100 m; 16 Jul. 1987; Josef Poelt leg.; GZU. • Oristano Prov., Catena del Marghine, Nuraghe Ortachis, Badde Salighe; 40°21'01"N, 08°54'24"E; 1020 m; 11 Mar. 2014; Nascimbene leg.; on *Quercusilex* in a forest site rich in *Lobariapulmonaria*; Na 4830.

A temperate to humid subtropical species found on subneutral bark of broad-leaved trees in open deciduous woodlands near rivers, very rarely calcicolous or muscicolous.

***Bacidinadelicata* (Leight.) V. Wirth & Vězda**

**Trentino-Alto Adige** • Trento Prov., Paneveggio-Pale di San Martino Natural Park, Val Ceremana; 46°17'21"N, 11°43'21"E; 1640 m; Sep. 2012; Nascimbene leg.; on *Piceaabies* in old growth forest; Na 3143. • Ibidem; 46°17'26"N, 11°43'14"E; 1780 m; Sep. 2012; Nascimbene leg.; on *Piceaabies* in old growth forest; Na 3144.

A mainly Mediterranean-Atlantic to humid subtropical species that was found in a very humid situation together with several other suboceanic lichens.

***Bacidinaegenula* (Nyl.) Vězda**

**Trentino-Alto Adige** • Trento Prov., Val di Fassa, Mazzin; 46°27'34"N, 11°42'00"E; 1400–1450 m; 07 Apr. 1979; Hafellner leg.; on dolomitic rocks in open forest habitat; GZU – Ha 4617.

A mild-temperate to humid subtropical species, most common on pebbles over moist ground in areas with siliceous substrata; certainly overlooked and probably more widespread in Tyrrhenian Italy, with outposts in the Alps.

***Bellemereasubsorediza* (Lynge) R. Sant.**

**Lombardia** • Brescia Prov., Adamello Natural Park, Passo del Tonale, on the north side of Monticello di Mezzo, below the funicular; 1950 m; 24 Jul. 2006; Hafellner and Muggia leg.; on granite; TSB 38540. **Third record for Italy.**

On siliceous rocks in open lichen communities near or above treeline (i.e. near glaciers). Known from Trentino-Alto Adige (Nimis 2016) and probably more widespread in the Alps, but overlooked, being mostly sterile.

***Blasteniagennargentuae* Vondrák**

**Calabria** • Reggio Calabria Prov., Aspromonte Massif, Pietra Impiccata; 1750 m; Summer 1988; Nimis and Tretiach leg.; on siliceous rocks; TSB 12205. **Second record for Italy.**

A recently-described species of siliceous rocks in the Mediterranean mountains. The record from Calabria (Aspromonte) was cited by Nimis (1993: 167) under *B.festivella*.

***Blasteniamonticola* Arup & Vondrák**

**Piemonte** • Cuneo Prov., Ligurian Alps above Colle Bertrand, W above Upega; 1960 m; Summer 2000; Nimis & Tretiach leg.; on conifers; TSB 33234.

A recently described species growing on acid bark, mostly of conifers, or on the branches of shrubs in the subalpine belt. Most previous records of *B.herbidella* from the Alps (see Nimis 1993, 2016) could refer to this species, which is probably widespread throughout the Alps.

***Blasteniapsychrophila* Halıcı & Vondrák**

**Veneto** • Belluno Prov., Casera Razzo; 1750 m; Summer 1981; Nimis leg.; on siliceous rocks; TSB 1678. – **Friuli Venezia Giulia** • Udine Prov., near the summit of Mt. Paularo; about 2000 m; Summer 2000; P. Fragiacomo and Nimis leg.; on siliceous rocks; TSB 1707.

A recently-described species growing on base-rich siliceous rocks in the southern and Central European mountains, mostly above or near treeline. Several earlier records of *B.crenularia* (see Nimis 1993, 2016) from alpine-subalpine situations probably refer to this species.

***Buelliaatrocinerella* (Nyl.) Scheid.**

**Toscana** • Livorno Prov., Arcipelago Toscano National Park, Elba island, Madonna di Monserrato; 42°47'02"N, 10°23'31"E; 120 m; 26 Nov. 1989; Mayrhofer and J. Sattler leg.; C. Scheidegger det.; on siliceous rock, radiolarite; GZU – Ma 8696. **Second record for Italy.**

A Mediterranean species of hard siliceous rocks in warm-dry habitats, sometimes growing on other crustose lichens.

***Caliciumadspersum* Pers.**

**Emilia-Romagna** • Bologna Prov., Vergato, Lizzano in Belvedere, Camugnano, Montese, Alto Reno Terme; 44°08'00"N, 10°53'01"E; 890 m; 2018; S. Gambini leg.; on old *Castaneasativa* trees in traditional orchards; Na 6012. • Ibidem; 44°13'28"N, 10°55'41"E; 900 m; S. Gambini leg.; Na 6013. • Ibidem; 44°13'17"N, 10°55'48"E; 950 m; S. Gambini leg.; Na 6018. • Ibidem; 44°07'58"N, 11°05'40"E; 860 m; S. Gambini leg.; Na 6022. • Ibidem; 44°08'00"N, 11°05'44"E; 870 m; S. Gambini leg.; Na 6014. • Ibidem; 44°12'54"N, 11°05'56"E; 870 m; S. Gambini leg.; Na 6015. • Ibidem; 44°08'07"N, 10°53'56"E; 670 m; S. Gambini leg.; Na 6016. • Ibidem; 44°13'45"N, 10°55'36"E; 890 m; S. Gambini leg.; Na 6017. • Ibidem; 44°19'45"N, 11°02'41"E; 840 m; S. Gambini leg.; Na 6018. • Ibidem; 44°07'55"N, 10°54'09"E; 600 m; S. Gambini leg.; Na 6019.

A holarctic, temperate species found on bark, rarely on wood of deciduous trees, especially *Quercus* sp., often in fissures of the bark, more rarely on conifers.

**Calogaya arnoldii (Wedd.) Arup, Frödén & Søchting subsp. arnoldii**

**Trentino-Alto Adige** • Trento Prov., Stenico, trail near the offices of the Adamello-Brenta Natural Park; 46°03'22"N, 10°50'25"E; 720 m; 08 Apr. 2021; Nascimbene leg.; on limestone; Na 7243.

A well-distinct taxon of the critical *C.saxicola*-complex, found on steeply inclined surfaces of calciferous rocks (limestone, dolomite, calcareous schists) in open habitats; certainly more widespread in Italy. For further details see Gaya et al. (2001).

***Calogayarouxii* (Gaya, Nav.-Ros. & Llimona) provisionally placed here, ICN art. 36.1b**

**Piemonte** • Cuneo Prov., Cottian Alps, W ridge of Mt. Nebin, c. 1 km E of Colle di Sempeyre; 2380 m; Summer 2000; Nimis and Tretiach leg.; on calciferous siliceous rocks; TSB 32977.

A species with an Alpine distribution, growing mainly on the top of calcareous boulders in sunny and nutrient-enriched sites, often with *C.biatorina* and *Rusavskiaelegans*. The Italian distribution is poorly known, as the species was frequently confused with *C.arnoldii*, but probably it is widespread throughout the calcareous Alps, and also occurs in the Central Apennines. All of the Italian records prior to 2008 were under *C.arnoldii* (see Nimis 2016).

***Caloplacacoccinea* (Müll. Arg.) Poelt**

**Valle d’Aosta** • Aosta Prov., Western Alps, Monte Bianco (Mont Blanc) group, Val Veny W of Courmayeur, ridge W above the Rifugio Elisabetta Soldini; 45°45'45"N, 06°50'15"E; 2250 m; 30 Jul. 2001; Hafellner leg.; on steep, N-exposed cliffs and boulders of Jurassic limestone; GZU – Ha 85852. – **Piemonte** • Cuneo Prov., Western Alps, Cottian Alps, crest SW above Colle dell’ Agnello; 44°40'54"N, 06°58'35"E; 2830 m; 25 Jul. 2000; Hafellner leg.; on outcrops of calcareous schists on steep slope exposed to the SE; GZU – Ha 59417.

The thallus of *Caloplacacoccinea* is somewhat variable, ranging from entirely immersed and uncoloured to semi-immersed with orange particles. Diagnostic are the semi-immersed to sessile, vivid red apothecia, a colour unique among the alpine caloplacoid lichens. The species, which does not belong to *Caloplacas.str.*, mostly settles on steeply inclined to vertical rock faces of limestone cliffs and outcrops from the lower alpine to the nival belt.

***Caloplacamarmorata* (Bagl.) Jatta**

**Toscana** • Livorno Prov., Island of Pianosa, east coast; 10 m; 22 Mar. 2005; Muggia leg.; on limestone near the sea; TSB 36720.

This lichen has a complex nomenclatural history: Nimis (2016) states that the type of *Callopismamarmoratum* Bagl. (MOD-TSB), the basionym of *Xanthocarpiamarmorata* (Bagl.) Frödén, Arup & Søchting, has nothing to do with a *Xanthocarpia*, clearly corresponding with the lichen called *Caloplacasubochracea* (M. Choisy & Werner) Clauzade & Cl. Roux, which, according to Roux & Coll. (2014), is not identical with *Blasteniasubochracea* (Wedd.) Arup, Søchting & Frödén. The species, which does not belong to *Caloplacas.str.* nor to *Blastenia*, and whose DNA analysis is pending, has a Mediterranean distribution on compact limestone, being locally very abundant in coastal situations, and extremely rare far from the coast. The colour of the thallus, based on which several infraspecific taxa were distinguished (see e.g. Roux et al. 2014), is variable depending on exposure to sunlight, and intermediate forms are frequent. A new name for the lichen called *Xanthocarpiamarmorata* auct. will be also proposed in a forthcoming paper.

***Caloplacaturkuensis* (Vain.) Zahlbr.**

**Veneto** • Belluno Prov., Dolomiti d’Ampezzo Natural Park, near Ra Stua; 46°36'53"N, 12°05'54"E; 1600 m; 27 Aug. 2005; Thor leg.; U. Arup conf.; on *Acerpseudoplatanus*, on a steep gravelly slope; GT19355, UPS.

A mild-temperate lichen found on old deciduous trees, often near the base of the trunks, often overlooked, or confused with *C.cerina* (Šoun et al. 2011). It differs from *C.monacensis* in the well-developed, areolate, rarely fertile thallus with a thick layer of small granules, although molecular data suggest that it could be a sorediate-blastidiate morph of *C.monacensis* (Vondrák *in litt*.).

***Candelariellakuusamoënsis* Räsänen**

**Veneto** • Belluno Prov., Dolomiti Bellunesi National Park, Erera-Brendol; 46°09'44"N, 11°58'57"E; 1710 m; 18 Oct. 2020; Nascimbene leg.; on a wooden fence near the hut; Na 7199.

A boreal-montane, poorly understood lichen growing on the top of poles and wooden fences, on plant debris and soil, more rarely on rocks in upland areas; certainly more widespread in the Alps. The delimitation of this species is problematic: most of the material distributed in exsiccata belongs to other species, and it is doubtful whether the material called “*C.kuusamoensis*” by Central and Southern European authors really corresponds to the type material, which in itself resembles a luxuriant *C.vitellina* growing on mosses (Westberg, *in litt.*).

***Cetreliamonachorum* (Zahlbr.) W.L. Culb. & C.F. Culb.**

**Veneto** • Belluno Prov., Dolomiti Bellunesi National Park, Mt. Avena, Col Melon; 46°02'39"N, 11°50'37"E; 1300 m; 27 Feb. 1997; Nascimbene leg.; on *Fagussylvatica*; Na 356. • Belluno Prov., Cansiglio Forest, Due Ponti; 46°06'39"N, 12°25'04"E; 1100 m; Jan. 2002; Nascimbene leg.; on *Salix* sp.; Na 357. • Ibidem; 1100 m; 18 Dec. 2004; G. Caniglia leg.; on *Fagussylvatica*; Na 5143. • Belluno Prov., Dolomiti Bellunesi National Park, Vette Feltrine, Soladen; 46°03'45"N, 11°50'38"E; 870 m; 16 Feb. 2020; Nascimbene leg.; on *Fagussylvatica*; Na 6959, 6961, 6962. • Belluno Prov., Dolomiti Bellunesi National Park, Vette Feltrine, Col dei Cavai; 46°04'23"N, 11°50'02"E; 1360 m; 21 Jan. 1994; Nascimbene leg.; on *Fagussylvatica*; Na 354. • Belluno Prov., Dolomiti Bellunesi National Park, Longarone, Cajada Forest; 46°14'02"N, 12°14'37"E; 1300 m; Jul. 2010; Nascimbene leg.; on *Abiesalba*; Na 2359. • Belluno Prov., Lamon, Senaiga valley near Chioè; 46°02'34"N, 11°42'39"E; 460 m; 17 Mar. 2013; Nascimbene leg.; on *Piceaabies*; Na 2979. – **Sardegna** • Nuoro Prov., Gennargentu National Park, Punta Salinas, Baunei (Ogliastra); 40°06'03"N, 09°41'39"E; 450 m; Jul. 2010; Nascimbene leg.; on *Juniperus*; Na 2712.

A species with the imbricaric acid syndrome (major) and perlatolic acid (minor), mainly found on the bark of broad-leaved trees, more rarely on conifers and silicicolous mosses in humid, old, mostly montane forests; probably the most common species of *Cetrelia* in Italy.

***Chaenothecabrachypoda* (Ach.) Tibell**

**Abruzzo** • Chieti Prov., Abetina di Rosello Natural Reserve; 41°52'36"N, 14°21'23"E; 1000 m; Jul. 2009; Nascimbene leg.; on wood; Na 2487.

On decorticated stumps of deciduous and coniferous trees, more rarely on bark and siliceous rocks in old humid forests, on faces slightly protected from rain. Included in the Italian red list of epiphytic lichens as “Endangered” (Nascimbene et al. 2013).

***Chaenothecabrunneola* (Ach.) Müll. Arg.**

**Emilia-Romagna** • Bologna Prov., Camugnano; 44°07'58"N, 11°05'40"E; 870 m; 2018; S. Gambini leg.; on old *Castaneasativa* trees in traditional orchards; Na 6519. • Ibidem; 44°08'00"N, 11°05'44"E; 860 m; 2018; S. Gambini leg.; Na 6520.

This species is included in the Italian red list of epiphytic lichens as “Near-threatened” (Nascimbene et al. 2013).

***Chaenothecaphaeocephala* (Turner) Th. Fr.**

**Emilia-Romagna** • Bologna Prov., Montese; 44°13'45"N, 10°55'36"E; 840 m; 2018; S. Gambini leg.; on old *Castaneasativa* trees in traditional orchards; Na 6020.

A cool-temperate, holarctic lichen mainly found on old *Quercus* sp. in open woodlands, in bark fissures seldom wetted by rain. Our specimen was collected on a very old *Castanea* tree near Bologna (Pezzi et al. 2020).

***Chaenothecastemonea* (Ach.) Müll. Arg.**

**Abruzzo** • Chieti Prov., Abetina di Rosello Natural Reserve; 41°52'54"N, 14°21'27"E; 940–1000 m; Jul. 2009; Nascimbene leg.; on *Abiesalba*; Na 2404. • Ibidem; 22–23 Oct. 2015; Nascimbene leg.; Na 4670, 4691, 4715.

A cool-temperate to boreal-montane, circumpolar lichen found in rain-protected hollows of conifer trunks inside forests, especially near the ground, both on bark and wood.

***Chaenothecatrichialis* (Ach.) Th. Fr.**

**Abruzzo** • Chieti Prov., Abetina di Rosello Natural Reserve; 41°52'54"N, 14°21'27"E; 1000 m; Jul. 2009; Nascimbene leg.; on *Abiesalba*; Na 2403.

A holarctic species found on acid-barked deciduous trees, conifers and wood in forests and woodlands; widespread in upland areas throughout the country, but most common in the Alps.

***Chaenothecopsisdebilis* (Sm.) Tibell**

**Trentino-Alto Adige** • Trento Prov., Paneveggio-Pale di San Martino Natural Park, Val Canali, Villa Welsperg; 46°11'57"N, 11°52'06"E; 1000 m; 31 May 2012; Nascimbene leg.; on old *Tilia* sp.; Na 2757.

This species was collected in the park surrounding an historical building, on the trunk of an old lime-tree that presented some wood areas protected from rain (i.e. dry conditions). The species was probably overlooked in Italy, as indicated by its current scattered distribution pattern across the country.

***Cladoniaarbuscula* (Wallr.) Flot.**

**Marche** • Fermo Prov., Montefortino; 1846; D. Marzialetti leg.; Gheza det.; BOLO – Herb. Bertoloni (as *C.rangiferina*).

A circumpolar, boreal-subarctic-subalpine lichen, one of the most abundant elements of lichen-rich tundra-like vegetation on mineral soil in exposed habitats. This specimen was likely collected in the summit area of the mountains near Montefortino where altitude exceeds 2000 m.

***Cladoniamitis* Sandst.**

**Marche** • Fermo Prov., Montefortino; no date [probably 1846]; D. Marzialetti leg.; Gheza det.; BOLO – Herb. Bertoloni (as *C.rangiferina*).

A typical member of subalpine-alpine tundras, perhaps more common at higher altitudes than *C.arbuscula*. This specimen was probably collected in the same stand of *C.mitis* (see above). This is the southernmost record for Italy.

***Diplotommachlorophaeum* (Leight.) Kr.P. Singh & S.R. Singh**

**Calabria** • Cosenza Prov., between Frascineto and Villapiana Scalo, along the Raganello river; 39°48'46"N, 16°19'54"E; 200 m; 04 Jun. 1979; Mayrhofer leg.; on rock; GZU – Ma 1142.

A temperate, perhaps holarctic early coloniser of basic siliceous rocks and roofing tiles; overlooked, and certainly more widespread in Italy.

***Diplotommalutosum* A. Massal.**

**Trentino-Alto Adige** • Trento Prov., Paneveggio-Pale di San Martino Natural Park, Castellazzo; 46°18'28"N, 11°47'56"E; 2270 m; 14 Sep. 2020; Nascimbene leg.; on carbonatic rocks; Na 7134. • Ibidem; 46°18'26"N, 11°47'43"E; 2330 m; 14 Sep. 2020; Nascimbene leg.; Na 7135. – **Veneto** • Belluno Prov., Dolomiti Bellunesi National Park, near Passo Falzarego; 46°30'49"N, 12°00'26"E; 2170 m; 26 Sep. 1985; Mayrhofer leg.; on carbonatic rock; GZU – Ma 8313. – **Basilicata** • Potenza Prov., Piana del Pollino, NW Serra delIe Ciavole; 39°55'09"N, 16°12'57"E; 1900 m; 02 Jun. 1979; Josef Poelt leg.; on rock; GZU – Po 1180.

An apparently widespread but rare, or at least rarely distinguished species, characterised by four-celled spores and a J+ blue medulla .

***Diplotommamurorum* (A. Massal.) Coppins**

**Trentino-Alto Adige** • Trento Prov., Paneveggio-Pale di San Martino Natural Park, Passo Rolle, near Baita Segantini; 46°17'56"N, 11°48'13"E; 2200 m; 14 Sep. 2020; Nascimbene leg.; on arenaceous calciferous rocks (Werfen-formation), parasitic on *Caloplacaerythrocarpa*; Na 7119.

A mild-temperate lichen starting the life-cycle on species of the *Caloplacateicholyta*-complex, the peculiar biology of which deserves further study. It was certainly overlooked in Italy, as indicated by its current scattered distribution pattern across the country. It is similar to *D.scheideggerianum* that has shorter spores and usually grows on *Leproplaca*-species.

***Dirommadirinellum* (Nyl.) Ertz & Tehler**

**Lazio** • Latina Prov., Borgo Montello; 41°30'28"N, 12°46'22"E; 30 m; Dec. 2011; Nascimbene leg.; on *Quercuscerris*; Na 2967. • Ibidem; 41°27'46"N, 12°47'18"E; 30 m; Dec. 2011; Nascimbene leg.; Na 2968. • Ibidem; 41°30'21"N, 12°45'48"E; 30 m; Dec. 2011; Nascimbene leg.; Na 2969.

A rare species living as a parasite on *Dirinaceratoniae*, strictly confined to the Mediterranean belt. Our specimen was collected on isolated trees in an agricultural landscape near the Tyrrhenian coast.

***Eigleraflavida* (Hepp) Hafellner**

**Liguria** • Imperia Prov., Western Alps, Alpi Liguri, mountain ridge S above the village Monesi, on the ridge W above Col Garezzo; 44°02'50"N, 07°46'25"E; 1850 m; 21 Jul. 2000; Hafellner leg.; on small outcrops of calcareous schist in subalpine pasture, on almost horizontal faces of low outcrops; GZU – Ha 84338.

A very inconspicuous species which, on account of its mostly ochraceous to grey thallus and minute black aspicilioid apothecia, is often detected only in screenings of larger collections on limestone pebbles or rock pieces of low outcrops from higher altitudes. Diagnostic are the blue-green epihymenium and asci with a distinct tholus reacting intensely blue with Lugol’s reagent, which is the distinguishing character from otherwise similar *Hymenelia*-species.

***Eiglerahomalomorpha* (Nyl.) Hafellner & Türk**

**Trentino-Alto Adige** • Trento Prov., Dolomites, Sass Becè, Pordoi; 46°28'50"N, 11°48'36"E; 2300 m; 25 Oct. 1984; Hafellner leg.; on a dolomitic rock; GZU. **Second record for Italy.**

This species is locally common in the Alps on limestone near the ground, such as on basal parts of steep cliffs in upland areas.

***Elixiaflexella* (Ach.) Lumbsch**

**Veneto** • Belluno Prov., Dolomiti d’Ampezzo Natural Park, Passo Tre Croci; 46°33'24"N, 12°12'55"E; 1750 m; 25 Sep. 1985; Mayrhofer leg.; GZU – Ma 8304. – **Friuli Venezia Giulia** • Udine Prov., Carnic Alps, Monte Tinisa; 46°25'00"N, 12°42'00"E; 1750 m; 19 Aug. 1994; Hafellner leg.; on wood; GZU – Ha 76953.

This species may easily be mistaken for a non-lichenised ascomycete with minute black, angular apothecia, as the thallus is regularly present as an endoxylic discontinuous crust only. Vertical faces of coniferous stumps in upper montane forests are the preferred ecological niche, the ecology being similar to that of *Caliciumtrabinellum*, which is present as accompanying species on the cited specimen from Friuli.

***Fellhanerasubtilis* (Vězda) Diederich & Sérus.**

**Veneto** • Belluno Prov., Feltre, Vincheto di Celarda Natural Reserve; 46°01'17"N, 11°58'37"E; 310 m; Aug. 2011; Nascimbene leg.; on *Populus* sp. in a riparian forest; Na 5397. **Second record for Italy.**

Our specimen was collected at the bottom of the Piave Valley along the river, in a particular microclimatic condition (very humid and cold in winter, and dry-warm in summer). The species is currently included in the Italian red list of epiphytic lichens as “Endangered” (Nascimbene et al. 2013).

***Flavoplacalimonia* (Nimis & Poelt) Arup, Frödén & Søchting**

**Toscana** • Livorno Prov., Island of Pianosa, road from the prison to the east coast; 10 m; 22 Mar. 2005; Muggia and Tretiach leg.; on calcareous rocks; TSB 36728.

This species, described from the calcareous cliffs along the coast of the Island of Marettimo, is also known from inland localities, and is certainly more widespread in Italy; earlier records might be under *Caloplacacitrina* s.lat.

***Frutidellacaesioatra* (Schaer.) Kalb**

**Friuli Venezia Giulia** • Udine Prov., Carnic Alps, Mt. Crostis; 46°34'20"N, 12°53'20"E; 2240 m; 17 Aug. 1994; Hafellner leg.; on saxicolous mosses; GZU – Ha 84341.

This species, characterized by mostly whitish-grey minute almost granular areoles and lead-grey to blackish convex immarginate apothecia, is found on cushions of bryophytes on siliceous boulders and outcrops, mostly on N-facing slopes, from treeline to the alpine belt.

***Frutidellafurfuracea* (Anzi) M. Westb. & M. Svensson**

**Friuli Venezia Giulia** • Udine Prov., Carnic Alps, Monte Tinisa; 46°25'00"N, 12°42'00"E; 1750 m; 19 Aug. 1994; Hafellner leg.; on *Larixdecidua*; GZU – Ha 76938.

Diagnostic for this species are the mostly brownish thallus with greenish-brownish, punctiform, flat, farinose soralia in combination with the lead-grey to blackish, convex, immarginate apothecia (recalling those of the type species of the genus, *F.caesioatra*, but larger and less convex). It grows on long-time moist sites from the montane belt to treeline, mostly on bark, more rarely on wood.

***Fuscopannariaignobilis* (Anzi) P.M. Jørg.**

**Molise** • Isernia Prov., Staffoli, near Vastogirardi; 41°45'36"N, 14°18'27"E; 1006 m; 23 Oct. 2015; Nascimbene leg.; on *Salixalba*; Na 4775.

This Mediterranean-Atlantic species was found in cracks of the bark of large trees (near the base of the boles) abundantly colonised by *Lobariapulmonaria*, along a tree row near a small river.

***Fuscopannariapraetermissa* (Nyl.) P.M. Jørg.**

**Valle d’Aosta** • Aosta Prov., Western Alps, Monte Bianco (Mont Blanc) group, Val Veny W of Courmayeur, ridge W above the Rifugio Elisabetta Soldini; 45°45'45"N, 06°50'15"E; 2250 m; 30 Jul. 2001; Hafellner leg.; on soil among cliffs and boulders of Jurassic limestone on a N-exposed slope; GZU – Ha 75410. – **Toscana** • Pistoia Prov., Northern Appenines, Abetone, Val di Luce, Alpe Tre Potenze; 44°07'30"N, 10°37'60"E; 1500–1820 m; 27 Oct. 1978; Hafellner leg.; on saxicolous bryophytes; GZU – Ha 4344.

An arctic-alpine to boreal-montane, circumpolar lichen found on calciferous soil, mosses and plant debris, with optimum near and above treeline. It is usually sterile, with dark-grey to brownish thalli mainly consisting of small, suberect squamules which likely also act as vegetative diaspores. In old herbarium specimens, the triterpenoids often crystallize into long, translucent needles resembling glassy hairs, a diagnostic character to distinguish this species from sterile thalli of *Massalongiacarnosa*.

***Gyalectaerythrozona* Lettau**

**Veneto** • Belluno Prov., Dolomiti Bellunesi National Park, Vette Feltrine, Passo delle Vette Grandi; 46°05'24"N, 11°50'37"E; 2010 m; 13 Jun. 2020; Nascimbene leg.; on a selciferous carbonatic rock (Formazione di Fonzaso); Na 7200. **Second record for Italy.**

A species of the *G.leucaspis*-group characterised by the entire (rather than radially incised) apothecial margins, and the elongate-fusiform (rather than acicular) ascospores. It is widespread in the Holarctic region, and in the Central European orobiomes it mostly occurs near or above treeline. In the Alps it seems to be most frequent in the eastern sector (Nimis et al. 2018a). Our specimen was collected on a steeply inclined, N-exposed rock in very moist, shaded, conditions, under overhangs.

***Gyalectafoveolaris* (Ach.) Schaer.**

**Friuli Venezia Giulia** • Udine Prov., Carnic Alps, Monte Bivera; 46°26'40"N, 12°37'45"E; 2250 m; 30 Jul. 1993; Hafellner leg.; terricolous in crevices; GZU – Ha 32694a. – **Veneto** • Belluno Prov., Dolomiti Bellunesi National Park, Vette Feltrine, near Passo delle Vette Grandi; 46°05'25"N, 11°50'34"E; 2030 m; 10 Apr. 2021; Nascimbene leg.; on terricolous bryophytes; Na 7159. – **Valle d’Aosta** • Aosta Prov., Western Alps, Monte Bianco (Mont Blanc) group, Val Veny W of Courmayeur, ridge W above the Rifugio Elisabetta Soldini; 45°45'45"N, 06°50'15"E; 2250 m; 30 Jul. 2001; Hafellner leg.; on soil; GZU – Ha 75411.

A circumpolar, arctic-alpine lichen occasionally found in the lower alpine belt of calcareous mountains, where it mostly settles on long-time humid, subvertical soil stripes. The ecology is similar to that of *Ramoniamelathelia* which may grow next on plant remnants.

***Gyalideafritzei* (Stein) Vězda**

**Trentino-Alto Adige** • Bolzano/Bozen Prov., Sciliar Natural Park, Bad Ratzes; 46°31'41"N, 11°35'03"E; 1300 m; 25 Aug. 2006; Nascimbene leg.; on siliceous rock along a creek; Na 4836. **Third record for Italy.**

This is an overlooked, but certainly not common silicicolous species typical of periodically inundated sites.

***Halecanialecanorina* (Anzi) M. Mayrhofer & Poelt**

**Trentino-Alto Adige** • Bolzano/Bozen Prov., Sciliar Natural Park, Castelrotto; 46°30'42"N, 11°35'17"E; 2200 m; 18 Jul. 2007; Nascimbene leg.; on plant debris among dolomitic rocks; Na 4167.

An often overlooked, but certainly uncommon species, perhaps more widespread in the Alps over calcareous substrata, with optimum near treeline.

***Heppiaadglutinata* (Kremp.) A. Massal.**

**Veneto** • Belluno Prov., Dolomiti d’Ampezzo Natural Park, Croda del Becco; 46°40'02"N, 12°04'56"E; 2400 m; 02 Aug. 1997; Nascimbene leg.; on calcareous soil; Na 641. • Belluno Prov., Dolomiti Bellunesi National Park, Vette Feltrine, Busa di Cavaren; 46°05'44"N, 17°49'40"E; 1950 m; 20 Nov. 1994; Nascimbene leg.; on calcareous soil; Na 642.

A cool-temperate to boreal-montane, circumpolar, ephemeral lichen of disturbed calciferous soil in dry, open grasslands.

***Hymeneliaheteromorpha* (Kremp.) Lutzoni**

**Lombardia** • Brescia Prov., Adamello Natural Park, Val Paghera di Vezza along the path from Rifugio alla Cascata to Rifugio Aviolo; 46°12'38"N, 10°24'49"E; 1800 m; 25 Jul. 2006; Thor leg.; , on mortar; UPS-L-166762.

A probably holarctic species found on dolomite and hard limestone in rather sheltered situations, with optimum near treeline, as in the case of our record.

***Hypotrachynaafrorevoluta* (Krog & Swinscow) Krog & Swinscow**

**Trentino-Alto Adige** • Trento Prov., Paneveggio-Pale di San Martino Natural Park, Val Canali; 46°13'06"N, 11°54'32"E; 1200 m; 08 Aug. 2013; Nascimbene leg.; on *Abiesalba*; Na 3807. **First record from the Italian Alps.**

This species, widely distributed on both Hemispheres, is very similar to *H.revoluta* (for the main differences see Masson 2005), and some Italian records of the latter could refer to it. It seems to be widespread along the northern side of the Alps (Clerc 2006; Nimis et al. 2018a).

***Lecaniacyrtellina* (Nyl.) Sandst.**

**Veneto** • Belluno Prov., Feltre, Villabruna; 46°03'05"N, 11°55'40"E; 350 m; 25 Jan. 2020; Nascimbene leg.; on *Malus* sp.; Na 6809. – **Abruzzo** • Chieti Prov., Abetina di Rosello Natural Reserve; 41°52'54"N, 14°21'27"E; 1000 m; Jul. 2009; Nascimbene leg.; on an isolated tree with *Athalliaalnetorum* and *Lecanorahoriza*; Na 2476.

A species found on the base-rich bark of more or less isolated deciduous trees, not always distinguished from *L.cyrtella* by Italian authors.

***Lecanoracavicola* Creveld**

**Trentino-Alto Adige** • Bolzano/Bozen Prov., Val di Roja, east ridge of the Outer Nockenkopf, just below the summit; 46°49'25"N, 10°27'50"E; 2700 m; 22 Apr. 1984; Hafellner leg.; on overhanging siliceous rock; GZU – Ha 12476. • Bolzano/Bozen Prov., Upper Venosta Valley/Vintschgau, NE slopes of the Elferspitze; 46°46'55"N, 10°29'35"E; 2650 m; 25 Jul. 2006; Hafellner leg.; on overhanging siliceous rock; GZU – Ha 12329. – **Lombardia** • Brescia Prov., Adamello Natural Park, Edolo, Passo Gallinera; 46°10'55"N, 10°24'45"E; 2340 m; 25 Jul. 2006; Hafellner leg.; on overhanging rock faces of cliffs of siliceous schist on the crest; GZU – Ha 85845.

This sorediate and only rarely fertile species has a peculiar chemistry (atranorin and alectorialic acid, Poelt and Leuckert 1984), the latter causing a reddish miscoloration of the freshly yellowish-ochre areoles after some time of storage in the herbarium. It is a characteristic colonizer of overhangs of siliceous rocks, from treeline high up in the alpine belt, where *Aspiciliamashiginensis*, *Lecanoraorbicularis*, *L.swartzii*, *Psoriniaconglomerata*, and *Sporastatiapolyspora* are among the accompanying species. It is fairly common in the siliceous mountains near the eastern border areas of the Alps, but apparently much rarer in the central and western parts.

**Lecanoraepibryon(Ach.)Ach.var.bryopsora Doppelb. & Poelt**

**Lombardia** • Brescia Prov., Adamello Natural Park, Passo del Tonale, Cima di Cadì, on the top and below on the south-east ridge; 46°16'33"N, 10°34'15"E; 2590 m; 28 Jul. 2006; Hafellner and Muggia leg.; on soil and plant debris; TSB 38476, GZU. – **Trentino-Alto Adige** • Bolzano/Bozen Prov., Dolomites, Puez-Odle Natural Park, Ortisei/St.Ulrich, M. Seceda; 46°36'05"N, 11°44'17"E; 2470 m; 02 Sep. 2002; Hafellner leg.; on crevices and plant debris; GZU.

This is a sorediate lichen known from Piedmont (Nimis et al. 2018a), but certainly more widespread in the Alps. Difficult to recognise, being often sterile, it is perhaps just a sorediate morph of *L.epibryon* (Nimis 2016).

***Lecanorahoriza* (Ach.) Linds.**

**Trentino-Alto Adige** • Trento Prov., Val del Merlo, Monte Bondone Natural Reserve; 46°00'25"N, 11°02'04"E; 1640 m; Oct. 2003; Nascimbene leg.; on *Fagussylvatica* in a thermophilous open stand; Na 4095.

A mainly Mediterranean species found on smooth bark of broad-leaved trees. It is rarer in the Alps than in the Mediterranean mountains, as indicated by the few records from northern Italy. Our specimen was collected on isolated *Fagussylvatica* trees.

***Lecanoralojkaeana* Szatala**

**Veneto** • Belluno Prov., Dolomiti Bellunesi National Park, Vette Feltrine, Passo delle Vette Grandi; 46°05'24"N, 11°50'37"E; 2010 m; 13 Jun. 2020; Nascimbene leg.; on a selciferous carbonatic rock under overhangs; Na 7201. **Second record for Italy.**

A rarely collected species known from the Alps, the Central European mountains and Scandinavia, found beneath underhanging surfaces of siliceous rocks in upland areas; perhaps overlooked and more widespread in the Alps, being almost always sterile. Our specimen was collected on flint nodules in overhanging rocks of the late Jurassic formation “Formazione di Fonzaso”.

***Lecanorasilvae-nigrae* V. Wirth**

**Lombardia** • Brescia Prov., Adamello Natural Park, Edolo, Passo Gallinera; 46°10'55"N, 10°24'45"E; 2340 m; 25 Jul. 2006; Hafellner leg.; on inclined siliceous schist faces; GZU – Ha 85842. – **Toscana** • Pistoia Prov., Abetone, Val di Luce, Alpe Tre Potenze; 44°07'60"N, 10°37'60"E; 1500–1820 m; 27 Oct. 1978; Josef Poelt leg.; on siliceous rock; TLC: usnic, protocetraric, rangiformic, norrangiformic (analysed by H. Vänskä); GZU.

This species recalls *L.alpigena*, a large-fruiting species of the *L.polytropa*-group, but apothecial discs and margins are more discolourous, the discs being mostly reddish-brown, and the margins react P+ orange due to protocetraric acid;.the apothecia are often arranged in scattered groups on a poorly developed thallus. It grows on siliceous boulders including metal-rich rocks, mostly in the upper montane to lower alpine belts.

***Lempholemmaintricatum* (Arnold) Zahlbr.**

**Veneto** • Belluno Prov., Dolomiti Bellunesi National Park, Vette Feltrine, near Passo delle Vette Grandi; 46°05'25"N, 11°50'34"E; 2030 m; 10 Apr. 2021; Nascimbene leg.; on marly limestone (Rosso Ammonitico Superiore); Na 7167.

On steeply inclined surfaces of calcareous or basic siliceous rocks in seepage tracks, mostly in humid areas, as in our site.

***Lepraschaereri* (Hafellner) Hafellner**

**Lombardia** • Brescia Prov., Adamello Natural Park, Edolo, Passo Gallinera; 46°10'49"N, 10°24'49"E; 2320 m; 16 Jun. 2004; Nascimbene leg.; on base-rich siliceous rock; Na 5390, 5392.

This species seems to be restricted to high-elevation sites of the Alps and the Apennines, reaching the nival belt.

***Leprateneriffensis* (Vain.) Hafellner**

**Sardegna** • Olbia-Tempio Prov., Archipelago della Maddalena, Island of Caprera; 41°12'22"N, 09°27'48"E; near sea level; 14 Apr. 2003; Tretiach leg.; T. Craighero and M. Tretiach rev.; on siliceous rocks; TSB 43336, 43337. • Olbia-Tempio Prov., Arcipelago della Maddalena, Island of Spargi; 41°12'33"N, 09°27'48"E; 14 Apr. 2003; Tretiach leg.; T. Craighero and M. Tretiach rev.; on siliceous rock; TSB 43335. **Second records from Italy.**

A Mediterranean-Macaronesian silicicolous species, for Italy previously known only from the island of Linosa.

***Leptogiumbyssinum* (Hoffm.) Nyl.**

**Lombardia** • Brescia Prov., Adamello Natural Park, Breno, Passo di Crocedomini, Val Fredda, Passo di Val Fredda, above the saddle on the south-east side of Mt. Frerone; 2340 m; 26 Jul. 2006; Hafellner and Muggia leg.; on soil over siliceous substrata; TSB 38503. **Second record from Italy.**

An inconspicuous, perhaps overlooked, ephemeral lichen, previously known in Italy only from Trentino-Alto Adige/Südtirol (Nimis 2016; Nimis et al. 2018a).

***Lobothalliaalphoplaca* (Wahlenb.) Hafellner**

**Emilia-Romagna** • Parma Prov., Gravene, Prato Grande, Borgotaro; 44°28'53"N, 09°46'18"E; 500 m; 26 Jun. 1980; G. Caniglia leg.; on siliceous rocks; Na 5385.

A widespread species with an apparently disjunct distribution in mountain areas of the Northern Hemisphere, found on compact siliceous rocks wetted by rain in upland areas. It is locally abundant in the Alps, rarer in the Apennines.

***Massalongiacarnosa* (Dicks.) Körb.**

**Trentino-Alto Adige** • Trento Prov., Paneveggio-Pale di San Martino Natural Park, Cima Bocche; 46°20'03"N, 11°44'45"E; 2100 m; 04 Sep. 2020; Nascimbene leg.; on bryophytes on porphyric rock, and directly on rock; Na 6910. • Trento Prov., Stelvio National Park, Val de la Mare, Bosco di Celvestré near Malga Prabon; 46°24'22"N, 10°41'40"E; 1780 m; 27 Jul. 2006; Thor leg.; on mosses over siliceous boulders; UPS-L-166815. – **Toscana** • Pistoia Prov., Abetone, Val di Luce, Alpe Tre Potenze; 44°07'30"N, 10°37'60"E; 1500–1820 m; 27 Oct. 1978; Josef Poelt leg.; on saxicolus bryophytes; GZU.

A circumpolar, arctic-alpine to boreal-montane lichen that at first sight is similar to *Fuscopannariapraetermissa*, but the thalli are mostly paler brown, often forming small rosettes, and the isidia-like lobules are less dense and mostly marginal. As the thalli are devoid of lichen substances, no extruding needle-like crystals develop with age, a diagnostic character to distinguish sterile herbarium specimens of both species under the dissecting microscope. Fertile thalli with red-brown, sessile apothecia are not rare, as in the specimen from Tuscany.

***Micareaglobulosella* (Nyl.) Coppins**

**Abruzzo** • Chieti Prov., Abetina di Rosello Natural Reserve; 41°52'36"N, 14°21'23"E; 940 m; 22 Oct. 2015; Nascimbene leg.; on *Abiesalba*; Na 5086, 5087.

A temperate to probably circumboreal-montane species found on bark of conifers and *Quercus* spp. in humid forests, more rarely on wood. Included in the Italian red list of epiphytic lichens as “Data Deficient” (Nascimbene et al. 2013).

***Miriquidicainstrata* (Nyl.) Hertel & Rambold**

**Lombardia** • Brescia Prov., Adamello Natural Park, Passo del Tonale towards Passo del Paradiso; 46°15'10"N, 10°34'45"E; 1950 m; 24 Jul. 2006; Hafellner & Muggia leg.; on inclined faces of big granitic boulders, parasitic on *Lecanoraalpigena*, *Lecideaconfluens*, *Rhizocarpongeographicum*; GZU – Ha 85837. – **Sardegna** • Sassari Prov., Mt. Limbara; 1250–1300 m; 07 May 1986; Josef Poelt leg.; parasitic on *Rhizocarpongeographicum*; GZU. • Ibidem; Mayrhofer leg.; GZU – Ma 6379.

This species is morphologically characterised by brown, slightly concave to flat, often compound areoles with paler greyish margins, recalling those of *M.intrudens*, but has aspicilioid apothecia, while the latter is sorediate. In early stages of development, it grows parasitically on a wide range of silicicolous crusts, including species of *Aspicilia*, *Lecanora*, *Lecidea* and *Rhizocarpon*. Later on, the lichenicolous behaviour may not be obvious. Preferred microhabitats are horizontal to slightly inclined rock faces of siliceous boulders, from the montane to the lower alpine belt.

***Mycobilimbiaepixanthoides* (Nyl.) Hafellner & Türk**

**Lombardia** • Como Prov., Lanzo d’Intelvi, Foresta Monte Generoso; 45°57'23"N, 09°01'33"E; 1097 m; 26 Oct. 2018; Gheza leg.; on *Acerpseudoplatanus*; Herb. Gheza. • Brescia Prov., Paisco Loveno, Foresta Legnoli; 46°03'29"N, 10°14'52"E; 1286 m; 04 Aug. 2019; Gheza leg.; on *Acerpseudoplatanus*; Herb. Gheza. • Brescia Prov., Cisano, Foresta Gardesana Occidentale, surroundings of Malga Lorina; 45°48'30"N, 10°39'39"E; 1333 m; 02 Aug. 2019; Gheza leg.; on *Fagussylvatica*; Herb. Gheza.

On mossy trunks of deciduous trees, more rarely on siliceous rocks, with a few records from the Italian Alps, probably locally overlooked. The three sites are located in the Prealps of Lombardy suggesting that the whole prealpine area of this region could potentially host this species. It was found in moist broadleaved forest stands.

***Mycocaliciumsubtile* (Pers.) Szatala**

**Abruzzo** • Chieti Prov., Abetina di Rosello Natural Reserve; 41°52'36"N, 14°21'23"E; 1000 m; Jul. 2009; Nascimbene leg.; on *Abiesalba*; Na 2410.

A saprophyte on dry, hard wood, especially of conifers, in open situations, mostly in the montane and subalpine belts.

***Naetrocymbefraxini* (A. Massal.) R.C. Harris**

**Lombardia** • Brescia Prov., Adamello Natural Park, Val Paghera di Vezza, along the path from Rifugio alla Cascata to Rifugio Aviolo; 46°12'06"N, 10°24'52"E; 1730 m; 25 Jul. 2006; Thor leg.; on *Sorbusaucuparia*; UPS-L-166798.

A mild-temperate species found on smooth bark of (mostly) deciduous trees; most probably non-lichenised.

***Opegraphavermicellifera* (Kunze) J.R. Laundon**

**Veneto** • Belluno Prov., Feltre, Villabruna; 46°03'00"N, 11°55'46"E; 385 m; 02 Dec. 2017 and 26 Nov. 2017; Nascimbene leg.; on *Juglansregia* and *Carpinusbetulus*; Na 5293, 5301. – **Abruzzo** • Chieti Prov., Abetina di Rosello Natural Reserve; 41°52'54"N, 14°21'26"E; 950 m; 23 Oct. 2015; Nascimbene leg.; on *Abiesalba*; Na 4716, 4721, 4722, 4763.

A mild-temperate lichen found on old trees in humid areas, especially near rivers, on faces seldom wetted by rain. In the Italian Alps it seems to be rare. In both regions our specimens were collected along a north exposed, deep valley, very close to a river.

***Pertusariaflavicans* Lamy**

**Veneto** • Belluno Prov., Dolomiti Bellunesi National Park, Vette Feltrine, near Passo delle Vette Grandi; 46°05'24"N, 11°50'37"E; 2010 m; 13 Jun. 2020; Nascimbene leg.; on selciferous carbonatic rocks; Na 6972. • Ibidem; 12 Jul. 2020; Nascimbene leg.; Na 6975. • Belluno Prov., Dolomites, Arabba, Porta Vescovo; 46°27'43"N, 11°53'12"E; 2400 m; 16 Apr. 1979; Hafellner leg.; on basic siliceous rocks; GZU – Ha 4587 (as host of *Sclerococcumsaxatile* under the name of the lichenicolous fungus).

This chemically variable species grows on lime-free but mineral-rich siliceous rocks, mostly on sheltered, steeply inclined surfaces. The specimens from Passo delle Vette Grandi belong to the chemotype with stictic acid in addition to the common thiophaninic acid, and were collected on flint nodules or decalcified strata in overhanging rocks of the late Jurassic formation “Formazione di Fonzaso”.

***Pertusariaglomerata* (Ach.) Schaer.**

**Valle d’Aosta** • Aosta Prov., Western Alps, Monte Bianco (Mont Blanc) group, Val Veny W of Courmayeur, ridge W above the Rifugio Elisabetta Soldini; 45°45'45"N, 06°50'15"E; 2250 m; 30 Jul. 2001; Hafellner leg.; on plant remains; GZU – Ha 75436.

A rather conspicuous species characterised by perithecioid apothecia sunken into yellowish-white, subglobose thalline warts reacting K+ yellow turning red, due to norstictic acid (Hanko 1983). It is regularly found encrusting bryophytes and plant debris over calciferous soil, mostly in the lower alpine belt.

***Phaeophysciapusilloides* (Zahlbr.) Essl.**

**Trentino-Alto Adige** • Trento Prov., Paneveggio-Pale di San Martino Natural Park, Val Canali, Villa Welsperg; 46°11'57"N, 11°52'06"E; 1000 m; 31 May 2012; Nascimbene leg.; on *Tilia* sp.; Na 2752.

A temperate species found on isolated deciduous trees with nutrient-rich bark, in montane valleys as in the case of our specimen.

***Placidiopsispseudocinerea* Breuss**

**Lombardia** • Brescia Prov., Adamello Natural Park, Edolo, Passo Gallinera; 46°10'49"N, 10°24'49"E; 2320 m; 16 Jun. 2004; Nascimbene leg.; on soil; Na 5388.

An arctic-alpine, circumpolar lichen growing on soil and on moribund bryophytes on siliceous, base-rich or slightly calciferous soil (e.g. on calcareous schist), with optimum near and above treeline. It is certainly widespread through the Italian Alps, and also occurs in the central Apennines.

***Placynthiumdolichoterum* (Nyl.) Trevis.**

**Veneto** • Belluno Prov., Dolomiti Bellunesi National Park, Vette Feltrine, near Passo delle Vette Grandi; 46°05'25"N, 11°50'34"E; 2030 m; 10 Apr. 2021; Nascimbene leg.; on marly limestone (Rosso Ammonitico Superiore); Na 7166.

A poorly known species of the *P.nigrum* complex growing in humid-sheltered situations near or above treeline on basic siliceous or slightly calciferous rocks, as in our collection site.

***Placynthiumfiliforme* (Garov.) M. Choisy**

**Veneto** • Belluno Prov., Dolomiti Bellunesi National Park, Vette Feltrine, Col dei Cavai; 46°04'23"N, 11°50'02"E; 1450 m; 15 Oct. 2001; Nascimbene leg.; on calcareous rock; Na 1259. • Belluno Prov., Dolomiti Bellunesi National Park, Vette Feltrine, Erera-Brendol; 46°09'39"N, 11°58'35"E; 1700 m; 18 Oct. 2020; Nascimbene leg.; on calcareous rock; Na 7029.

A Mediterranean (-montane) to mild-temperate lichen growing on steeply inclined seepage tracks of calcareous rocks, with a rather wide altitudinal range. Our specimens were collected on the Jurassic “Calcari Grigi” and “Rosso Ammonitico Superiore” formations.

***Polysporinaurceolata* (Anzi) Brodo**

**Veneto** • Belluno Prov., Carnic Alps, M. Tiarfin; 46°28'10"N, 12°35'50"E; 2200 m; 27 Jul. 1993; Hafellner leg.; on calcareous rocks; GZU – Ha 32725.

A rarely recorded species. The taxonomic value of the genus *Polysporina* is so far unresolved (Westberg et al. 2015) and the generic placement of the species treated here appears to be provisional.

***Porinabyssophila* (Körb. ex Hepp) Zahlbr.**

**Veneto** • Belluno Prov., Dolomiti Bellunesi National Park, Vette Feltrine, Colle Cesta; 46°05'24"N, 11°50'37"E; 2010 m; 13 Jun. 2020; Nascimbene leg.; on selciferous calcareous rocks (Formazione di Fonzaso); Na 6829.

A mild-temperate to humid subtropical species found on calcareous rocks in damp and shaded habitats. Our specimen was collected on an N-exposed selciferous carbonatic rock in very moist, shaded conditions, together with other species with a trentepohlioid photobiont (e.g. *Dirinamassiliensis*, very abundant, *Gyalectaerythrozona*, and *G.hypoleuca*).

***Protoblasteniaaurata* Poelt & Vězda**

**Trentino-Alto Adige** • Trento Prov., Paneveggio-Pale di San Martino Natural Park; 46°18'28"N, 11°47'48"E; 2270 m; 14 Sep. 2020; Nascimbene leg.; on compact calcareous rocks; Na 7078. **Third record for Italy.**

A rarely collected species of calciferous rocks in upland areas. Its distribution in Italy, as well as in the Alps, is currently poorly known (Nimis et al. 2018a).

***Protoblasteniaterricola* (Anzi) Lynge**

**Valle d’Aosta** • Aosta Prov., Western Alps, Monte Bianco (Mont Blanc) group, Val Veny W of Courmayeur, ridge W above the Rifugio Elisabetta Soldini; 45°45'45"N, 06°50'15"E; 2250 m; 30 Jul. 2001; Hafellner leg.; on soil in open alpine vegetation; GZU – Ha 84337.

This is the terricolous counterpart of the closely related saxicolous *P.siebenhaariana*, indicated by a sister position of both taxa in a phylogenetic reconstruction (Ekman and Blaalid 2011). It occasionally transgrades from mineral-rich soil layers covering strongly weathered calciferous rocks to solid rock, and then distinguishing both taxa may be difficult.

**Protoparmeliopsismuralisvar.dubyi (Müll. Arg.) Hafellner & Türk**

**Veneto** • Belluno Prov., Dolomiti Bellunesi National Park, Vette Feltrine, Erera-Brendol; 46°09'44"N, 11°58'57"E; 1710 m; 18 Oct. 2020; Nascimbene leg.; on a wooden fence near a hut; Na 7010. • Belluno Prov., Dolomites, Arabba, Porta Vescovo; 46°27'43"N, 11°53'12"E; 2400 m; 16 Apr. 1979; Hafellner leg.; on basic siliceous rock; GZU – Ha 4600.

This lichen usually grows on weakly calciferous or base-rich siliceous rocks in upland areas, with optimum near and above treeline, but it may also occur on wood, as in the case of our collection. In the Italian Alps it is fairly common.

***Pseudothelommaocellatum* (Körb.) M. Prieto & Wedin**

**Veneto** • Belluno Prov., Dolomites, Croda da Lago; 46°29'32"N, 12°06'11"E; 2100 m; 08 Oct. 2006; D. Cester leg.; on a decaying stump of *Larix*; Na 1786.

A circumboreal-montane species growing on hard rotting wood, e.g. on poles and fences, more rarely on *Larix* and *Pinuscembra* in the subalpine belt. This floristic note rectifies the erroneous attribution to Trentino-Alto Adige by Nascimbene et al. (2008).

**PsoromatenueHenssenvar.boreale Henssen**

**Veneto** • Belluno Prov., Dolomiti d’Ampezzo Natural Park, Foses; 46°38'23"N, 12°05'54"E; 2100 m; Aug. 2002; Nascimbene leg.; on soil; Na 1339, 1340.

An arctic-alpine, circumpolar lichen weak in competition, found on wet, naked soil, near glaciers or late snow-beds over acidic substrata.

***Psorotichialugubris* (A. Massal.) Arnold**

**Friuli Venezia Giulia** • Udine Prov., Julian Pre-Alps, high Torre-Valley above Tanataviele, gorge of Rio Zaturran; about 750 m; 28 Nov. 1992; V. Calatayud and M. Tretiach leg.; on limestone in shade; TSB 16661. **Second record for Italy.**

Perhaps related to *P.murorum*, but with a verruculose to squamulose thallus and inconspicuous apothecia which are at first immersed, later prominent, and somewhat wider ascospores; overall distribution poorly known, with a few scattered records from the Alps.

***Puncteliajeckeri* (Roum.) Kalb**

**Sardegna** • Nuoro Prov., Gennargentu National Park, Punta Salinas, Baunei, Ogliastra; 40°06'03"N, 09°41'39"E; 450 m; Jul. 2010; Nascimbene leg.; on *Juniperus*; Na 2652.

This recently-resurrected species was often not distinguished from *P.subrudecta* in earlier studies. The predominantly marginal soralia and pruinose lobe tips are diagnostic. It grows on bark of isolated deciduous trees and is certainly widespread throughout Italy, including Mediterranean regions.

***Pyrenodesmiaerodens* (Tretiach, Pinna & Grube) Søchting, Arup & Frödén**

**Sicilia** • Palermo Prov., Madonie Mnts., top of Pizzo Carbonara; 1979 m; 17 Aug. 2011; L. Muggia and Ha. Weingartmann leg.; on limestone; TSB 42254.

This specis grows on exposed, subvertical faces of limestone and dolomite, including old monuments, in dry sites of the montane and subalpine belts. The total distribution extends to the Irano-Turanian Region.

***Ramalinasubgeniculata* Nyl.**

**Abruzzo** • Chieti Prov., Abetina di Rosello Natural Reserve; 41°52'57"N, 08°21'16"E; 990 m; 22 Oct. 2015; Nascimbene leg.; on *Quercuscerris*; Na 4489, 4493.

A Mediterranean-Macaronesian species mainly found on twigs of shrubs and young trees in warm-humid Mediterranean areas. Our specimen was collected in a humid *Quercuscerris* forest.

***Ramoniamelathelia* (Nyl.) Ertz**

**Valle d’Aosta** • Aosta Prov., Western Alps, Monte Bianco (Mont Blanc) group, Val Veny W of Courmayeur, ridge W above the Rifugio Elisabetta Soldini; 45°45'45"N, 06°50'15"E; 2250 m; 30 Jul. 2001; Hafellner leg.; on plant remains; GZU – Ha 75426.

This species is regularly found on plant remnants in *Caricionfirmae*–communities, mostly in humid, N-exposed sites. The ecology is similar to that of *Gyalectafoveolaris*, which has also been observed in the collection site.

***Rhizocarponatroflavescens* Lynge**

**Veneto** • Belluno Prov., Dolomiti Bellunesi National Park, Vette Feltrine, Colle Cesta; 46°05'24"N, 11°50'37"E; 2010 m; 13 Jun. 2020; Nascimbene leg.; on selciferous calcareous rocks (Formazione di Fonzaso); Na 6831, 6833.

A cool-temperate to arctic-alpine species growing on steeply inclined surfaces of base-rich, or weakly calciferous siliceous rocks. In Italy it is common in the subalpine and alpine belts, especially in the Alps. In our collection site it was abundant.

***Rhizocarponfurax* Poelt & V. Wirth**

**Piemonte** • Torino Prov., Mountains W of Pinerolo, northeastern slopes and ridges of the Punta Cialáncia; 44°52'60"N, 07°07'20"E; 2350 m; 26 Jul. 2001; Hafellner leg.; on siliceous boulders, parasitic on *Lecidealapicida* agg.; GZU – Ha 69377, 69378.

An invader of the thalli of *Lecidealapicida* s lat. It may be distinguished from the macroscopically similar *R.geographicum* by the frequently angular apothecia with at least partly rough, umbonate to subgyrose discs, and the soon pigmented, mostly 3-septate ascospores. It is widespread in the siliceous Alps, mainly in the lower alpine belt, but apparently it was often overlooked.

***Rhizocarponnorvegicum* Räsänen**

**Trentino-Alto Adige** • Trento Prov., Paneveggio-Pale di San Martino Natural Park, Cima Bocche; 46°20'48"N, 11°45'30"E; 2560 m; 13 Sep. 2020; Nascimbene leg.; porphyritic blocks of a military trench of WW1; Na 7202. • Bolzano/Bozen Prov., Melago, Vallelunga; 46°49'53"N, 10°39'57"E; 1920 m; Hafellner leg.; on iron-containing rocks; GZU – Ha 12395 (2 specimens). • Bolzano/Bozen Prov., Rojental, east ridge of the Outer Nockenkopf, just below the summit; 46°49'25"N, 10°27'50"E﻿; 2700 m; 22 Apr. 1984; Hafellner leg.; on iron-containing rocks; GZU – Ha 12473. **Second records for Italy.**

A pioneer species of schistose, slightly calciferous or basic eruptive rocks in upland areas, which occasionally starts the life-cycle on members of Acarosporaceae, e.g. *Acarosporasinopica* when on metal-rich schists.

***Rinodinaalbana* (A. Massal.) A. Massal.**

**Sicilia** • Messina Prov., Nebrodi National Park, Cesarò, along the road to Monte Soro, 0.8 km WSW of the top; 37°55'43"N, 14°41'08"E; 1740 m; 05 May 2012; J. Malíček, I. Frolov and J. Vondrák leg.; *Fagussylvatica* forest, on *Fagussylvatica*; Herb. Malíček 7589.

A temperate species found on isolated deciduous trees with more or less smooth bark.

***Rinodinaaspersa* (Borrer) J.R. Laundon**

**Piemonte** • Vercelli Prov., Borgosesia, basal part of M. Fenera; 22 May 1876; A. Carestia leg.; on porphyric rocks; RO (as *Rinodinatrachytica*). **Third record for Italy.**

On hard siliceous rocks near the ground in cold-humid habitats, sometimes on walls, mostly below the montane belt.

***Rinodinacapensis* Hampe**

**Veneto** • Belluno Prov., Dolomites, Passo Tre Croci; 46°33'24"N, 12°11'46"E; 1720–1780 m; 25 Sep. 1985; Mayrhofer leg.; on *Piceaabies*; GZU – Ma 8298.

A cool-temperate to boreal-montane pioneer species, mostly found on smooth bark, but also on wood, with optimum in the subalpine and montane belts.

***Rinodinacastanomela* (Nyl.) Arnold**

**Veneto** • Belluno Prov., Dolomites, saddle of M. Sief, Col di Lana; 46°30'23"N, 11°57'19"E; 2200 m; 30 Aug. 1981; T. Feuerer leg.; on sand-lime rocks (Wengen formation, middle Triassic); Herb. Feuerer 12181, HBG. **Second record for Italy.**

An arctic-alpine to boreal-montane, perhaps circumpolar lichen found under overhanging cliffs of weakly calcareous or basic siliceous rocks, marl and calciferous schist near or above treeline.

***Rinodinacastanomelodes* H. Mayrhofer & Poelt**

**Friuli Venezia Giulia** • Udine Prov., Carnic Alps, near Casera Novarzutta, north of Lateis, Sauris; 1600 m; 10 Sep. 1987; Josef Poelt leg.; on calcareous rocks; GZU.

An arctic-alpine to boreal-montane, perhaps circumpolar lichen found on limestone, marl and calcareous schists at and above treeline; widespread but not common in the Alps, where it can reach the nival belt, and also reported from the mountains of southern Italy.

***Rinodinacretica* H. Mayrhofer**

**Sicilia** • Palermo Prov., Madonie, slopes above Geraci Siculo, SW Castelbuono; 1200 m; Jun. 1988; M. Pietschmann leg.; on calcareous rocks; M. **Second record for Italy.**

A Mediterranean calcicolous species, probably somehow more widespread in southern Italy.

***Rinodinadubyana* (Hepp) J. Steiner**

**Liguria** • Imperia Prov., Ligurian Alps, Monte de la Guardia; 1600 m; 14 Sep. 1970; H. Wunder leg.; on rock; M 1345.

A mainly temperate species found on steeply inclined to underhanging, sunny surfaces of limestone and dolomite wetted by rain, sometimes also on pebbles on the ground, with optimum below the subalpine belt.

***Rinodinaluridescens* (Anzi) Arnold**

**Liguria** • Genova Prov., “in montibus di Reppia”; Caldesi leg.; MOD.

A Mediterranean-Atlantic lichen described from Tuscany, found on hard siliceous rocks subject to frequent humid winds, often near the coast; not uncommon in some parts of Mediterranean Italy.

***Rinodinaobnascens* (Nyl.) H. Olivier**

**Trentino-Alto Adige** • Bolzano/Bozen Prov., Venosta valley/Vintschgau, near Lasa; 900 m; 05 Sep. 1992; Hafellner leg.; on siliceous rocks; GZU – Ha 30594.

A Mediterranean-Atlantic lichen found on weakly inclined to horizontal surfaces of siliceous rocks wetted by rain, starting the life-cycle especially on *Aspiciliellaintermutans*, but sometimes on other lichens, e.g. *Rhizocarpon*-species.

***Rinodinaoleae* Bagl.**

**Emilia-Romagna** • Parma Prov., Parma, Strada Ugozzolo, Case Nuove; 44°49'51"N, 10°20'21"E; 45 m; 03 Mar. 2015; Nascimbene leg.; on *Tilia* sp.; Na 4898, 4899.

A submediterranean-Mediterranean epiphytic lichen which was overlooked or confused with similar species in the past. Our specimen was collected in an urban environment of the Po-Plain.

***Rinodinaolivaceobrunnea* C.W. Dodge & G.E. Baker**

**Calabria** • Reggio Calabria Prov., Aspromonte, Pietra Impiccata; 1700–1750 m; 12 Jul. 1988; Josef Poelt leg.; GZU.

An arctic-alpine, circumpolar species found on soil, bryophytes and plant debris in tundra-like environments over siliceous substrata; certainly widespread throughout the Alps, and also reported from the high Mediterranean mountains.

***Rinodinapityrea* Ropin & H. Mayrhofer**

**Calabria** • Catanzaro Prov., Serre di Catanzaro, Serra S. Bruno; 38°34'36"N, 16°19'45"E; 780 m; 14 Jul. 1988; R. Türk leg.; on *Populus* sp.; Herb. Türk 10040.

A temperate species found on asbestos-cement and mortar, often on walls, more rarely on dust-impregnated bark; easy to overlook, being often sterile.

***Rinodinaroscida* (Sommerf.) Arnold**

**Friuli Venezia Giulia** • Udine Prov., Carnic Alps, M. Tinisa, near the summit; 46°24'48"N, 12°43'07"E; 2100 m; 29 Jul. 1993; Hafellner leg.; plant debris in crevices; GZU – Ha.

An arctic-alpine, circumpolar species found on soil, bryophytes and plant debris over calcareous substrata in tundra-like habitats; widespread throughout the Alps.

***Rinodinateichophila* (Nyl.) Arnold**

**Emilia-Romagna** • Parma Prov., M. Testanello; Aug. 1899; C. Zanfrognini leg.; on rock; MOD. • Parma Prov., M. Santa Donna; Aug. 1899; C. Zanfrognini leg.; on rock; MOD. • Bologna Prov., Lizzano, Fiammineda; Aug. 1896; C. Zanfrognini leg.; on rock; MOD.

A widespread species growing on base-rich siliceous rocks, mostly on more or less calciferous sandstone, especially in nutrient-enriched situations such as on walls, tiles, brick or gravestones, mostly below the montane belt, also found in large conurbations.

***Rinodinatephraspis* (Tuck.) Herre**

**Lombardia** • Sondrio Prov., near Liro river; 1100 m; M. Anzi leg.; on mica schist (*Ad saxa micaceo-schistosa juxta flumen Liri*); UPS, Anzi: Lich. rar. Lang. exs. 561 (as *Rinodinaconfragosa*). **Second record for Italy.**

This exsiccatum was erroneously cited by Mayrhofer and Poelt (1979) and Mayrhofer (1984) under *Rinodinaarnoldii*. The species grows on siliceous rocks in upland areas, in moist and often shaded situations such as near waterfalls, rapids, gorges and shores of lakes, often associated with Cyanobacteria (*Stigonema*).

***Rinodinaturfacea* (Wahlenb.) Körb.**

**Veneto** • Belluno Prov., Carnic Alps, near the saddle between Col Marende and M. Tiarfin; 2000 m; 27 Jul. 1993; Hafellner leg.; on mosses and plant debris; GZU – Ha 32752.

An arctic-alpine, circumpolar lichen found on soil rich in humus and plant remains in tundra-like habitats.

***Rinodinelladubyanoides* (Hepp) H. Mayrhofer & Poelt**

**Puglia** • Foggia Prov., Gargano, Monte Saraceno; 41°41'48"N, 16°03'04"E; 100 m; 25 May 1972; H. Wunder leg.; on calcareous rock; M.

A mild-temperate to Mediterranean species found on hard, compact calcareous rocks, mostly on steeply inclined faces wetted by rain.

***Rostaniaceranisca* (Nyl.) Otálora, P.M. Jørg. & Wedin**

**Trentino-Alto Adige** • Bolzano/Bozen Prov., Dolomiti di Sesto Natural Park, Tre Cime di Lavaredo; 46°37'14"N, 12°17'13"E; 2354 m; 19 Sep. 2020; Nascimbene leg.; on calcareous soil; Na 7007. **Third record for Italy.**

An arctic-alpine lichen that typically grows over frost-disturbed, weakly calcareous soil above treeline, as in the case of our collection site.

***Sarcogynefallax* H. Magn.**

**Trentino-Alto Adige** • Bolzano/Bozen Prov., Passo della Mendola; 30 Apr. 1965; Josef Poelt leg.; on rock; GZU.

A mainly mild-temperate lichen found on steeply inclined to underhanging surfaces of base-rich siliceous rocks, more rarely on calcareous rocks.

***Scutulacircumspecta* (Vain.) Kistenich, Timdal, Bendiksby & S. Ekman**

**Veneto** • Belluno Prov., Feltre, Vincheto di Celarda Natural Reserve; 46°00'49"N, 11°58'37"E; 310 m; 2005; Nascimbene leg.; on *Sambucusnigra*; Na 2326.

A mild-temperate lichen growing on old trees in open, humid woodlands below the subalpine belt, more rarely on primarily acid, but nutrient-enriched bark. Our material was collected in a humid riparian forest.

***Scytiniumaragonii* (Otálora) Otálora, P.M. Jørg. & Wedin**

**Veneto** • Belluno Prov., Dolomiti Bellunesi National Park, Vette Feltrine, Cordin delle Vette, Busa delle Vette; 46°05'17"N, 11°51'06"E; 1950 m; 31 Oct. 2020; Nascimbene leg.; on pleurocarpous mosses in rock crevices at the base of a N-exposed calcareous wall; Na 7057. • Ibidem; 46°05'20"N, 11°51'09"E; 1950 m; 13 Jun. 2021; Nascimbene leg.; Na 7231.

A recently-described species, widespread throughout Europe growing on pleurocarpous mosses close to the base of trunks, over mossy walls or calcareous rocks within forests, or on mosses in rock fissures within dry subalpine grasslands.

***Scytiniumimbricatum* (P.M. Jørg.) Otálora, P.M. Jørg. & Wedin**

**Trentino-Alto Adige** • Bolzano/Bozen Prov., Sciliar Natural Park, Tuffal, Fiè; 46°30'18"N, 11°32'51"E; 1700 m; Jul. 2006; Nascimbene leg.; on terricolous mosses and soil in alpine grasslands; Na 4285. • Bolzano/Bozen Prov., Sciliar Natural Park, Rifugio Bolzano; 46°30'33"N, 11°34'00"E; Jul. 2007; Nascimbene leg.; on terricolous mosses and soil in alpine grasslands; Na 3033. – **Veneto** • Belluno Prov., Dolomiti Bellunesi National Park, Vette Feltrine, Colle Cesta, near Rifugio Dal Piaz; 46°05'24"N, 11°50'37"E; 2010 m; 13 Jun. 2021; Nascimbene leg.; on terricolous mosses; Na 7234.

This species seems to be bound to high elevations and is likely to be widespread in the Alps, as well as in the higher mountains of the Apennines.

***Scytiniummassiliense* (Nyl.) Otálora, P.M. Jørg. & Wedin**

**Veneto** • Belluno Prov., Feltre, Rocchetta di San Vittore; 46°00'11"N, 11°56'43"E; 400 m; 16 May 2021; Nascimbene leg.; on limestone; Na 7216.

A mild-temperate to Mediterranean species found on steeply inclined surfaces of calcareous rocks with periodical seepage of water.

***Sphinctrinaleucopoda* Nyl.**

**Abruzzo** • Chieti Prov., Abetina di Rosello Natural Reserve; 41°52'57"N, 08°21'16"E; 1000 m; Jul. 2009; Nascimbene leg.; parasitic on *Lepraalbescens* on *Quercuscerris*; Na 2405, 2406.

A non-lichenized parasite on the thalli of epiphytic crustose lichens, certainly declining.

***Stictalimbata* (Sm.) Ach.**

**Trentino-Alto Adige** • Trento Prov., Val Noana; 46°07'52"N, 11°50'31"E; 1200 m; 23 Jul. 2014; Nascimbene leg.; on *Fagussylvatica* in a mixed, humid *Fagussylvatica*-*Abiesalba* forest; Na 4430.

A humid subtropical to Mediterranean-Atlantic species growing on bark, often associated with bryophytes, on mossy rocks and soil in very humid situations, certainly worthy of protection in Italy, being included in the Italian red list of epiphytic lichens as “Vulnerable” (Nascimbene et al. 2013). In Northern Italy, it seems to be restricted to the eastern Alps where it is extremely rare.

***Thelidiumdionantense* (Hue) Zschacke**

**Veneto** • Belluno Prov., Dolomiti Bellunesi National Park, Vette Feltrine, near Passo delle Vette Grandi; 46°05'25"N, 11°50'34"E; 2010 m; 09 May 2021; Nascimbene leg.; on marly limestone (Rosso Ammonitico Superiore); Na 7227. • Belluno Prov., Dolomiti Bellunesi National Park, Vette Feltrine, Colle Cesta, near Rifugio Dal Piaz; 46°05'24"N, 11°50'37"E; 2010 m; 13 Jun. 2021; Nascimbene leg.; on marly limestone (Rosso Ammonitico Superiore); Na 7228.

On steeply inclined surfaces of calciferous rocks in upland areas, with several scattered records throughout the Alps (outside the Italian territory); known from the Central Apennines, probably more widespread in the Italian Alps.

***Thelidiumzwackhii* (Hepp) A. Massal.**

**Trentino-Alto Adige** • Trento Prov., Dolomites, Boè Group, Antersass; 46°31'12"N, 11°48'48"E; 2710 m; 24 Jul. 2019; Nascimbene leg.; on humid mineral soil; Na 6896.

A mainly temperate, ecologically broad-ranging pioneer species found on both calcareous and siliceous rocks and on thin layers of soil, occasionally also in periodically submerged sites. Our specimen was collected on mineral, poorly developed sandy soil abundantly colonised by *Carexbicolor*, indicative of periodically inundated conditions.

***Thelocarponlichenicola* (Fuckel) Poelt & Hafellner**

**Veneto** • Belluno Prov., Dolomites, Croda da Lago; 46°29'32"N, 12°06'11"E; 2100 m; 08 Oct. 2006; D. Cester leg.; on *Placynthiellauliginosa* on wood in *Larix*-forest; Na 1939.

On clay soil in disturbed sites, often in *Calluna*-heaths, doubtfully lichenised.

***Varicellariarhodocarpa* (Körb.) Th. Fr.**

**Valle d’Aosta** • Aosta Prov., Western Alps, Monte Bianco (Mont Blanc) group, Val Veny W of Courmayeur, ridge W above the Rifugio Elisabetta Soldini; 45°45'45"N, 06°50'15"E; 2250 m; 30 Jul. 2001; Hafellner leg.; on plant remains; GZU – Ha 75427 (fertile material).

Alpine thalli are usually thick, whitish pustulate, virtually sterile crusts reacting C+ red. The pustules soon become rough (sorediate state) and later rather frequently contain inconspicuous (often slightly pink), immersed ascomata, indicating that in some genera (e.g., *Varicellaria*, *Lepra*) soralia may be derived from ascomata. In the Alps, the species is mostly a coloniser of plant remains in alpine mats over acidic soils, with a preference for wind-exposed ridges. Over superficially decalcified substrata it may also occur on limestone or calcareous schists, as in the case of our site.

***Variosporaaustralis* (Arnold) Arup, Søchting & Frödén**

**Basilicata** • Potenza Prov., Piana del Pollino, NW Serra delle Ciavole; 39°55'09"N, 16°12'57"E; 1850 m; 02 Jun. 1979; Mayrhofer leg.; on calcareous rocks; GZU – Ma 21936. • Potenza Prov., Mt. Pollino, near Bosco di Chiaromonte; 1700 m; 02 Jun. 1979; Hafellner leg.; on calcareous rocks; GZU – Ha 4696.

On exposed calciferous rocks near or above treeline, e.g. on the top of large, isolated boulders and on steeply inclined to vertical surfaces.

***Verrucariapraetermissa* (Trevis.) Anzi**

**Veneto** • Belluno Prov., Feltre, Torbiera di Lipoi; 46°02'15"N, 11°57'23"E; 320 m; 20 Apr. 2021; Nascimbene leg.; on periodically submerged calcareous stones along a small creek near the peat bog; Na 7171.

A probably circumboreal freshwater species, submerged only for very short periods, mostly found along creeks, on mineral-rich siliceous rocks, more rarely on calcareous substrata.

***Violellafucata* (Stirt.) T. Sprib.**

**Friuli Venezia Giulia** • Udine Prov., Carnic Alps, Sauris Lake, Bosco della Stua; 46°26'35"N, 12°42'50"E; 1100 m; 16 Aug. 1994; Hafellner leg.; on *Alnusincana*; GZU – Ha 84339.

This species forms sterile thalli with whitish convex areoles reacting K+ yellow, P+ orange-red and UV- (due to the presence of atranorin and fumarprotoetraric acid), and becoming apically sorediate with coarse soredia, the outermost ones often being slightly bluish. Frequently the thalli are parasitized by *Tremellalichenicola* and the presence of its galls is a good hint as to the identity of the host. *V.fucata* colonises both the bark of a wide range of trees and wood (e.g. rotting snags), in Central Europe from the colline to the montane belt.

***Xylographapallens* (Nyl.) Harm.**

**Veneto** • Belluno Prov., Croda da Lago; 46°29'32"N, 12°06'11"E; 2100 m; 08 Oct. 2006; D. Cester leg.; on a stump; Na 1933, 3052.

Widespread in the Northern Hemisphere on wood, especially in exposed habitats becoming dry in summer, mainly in montane to subalpine coniferous forests, with a few scattered records from the Alps (Nimis et al. 2018a). The samples collected on a slightly decomposed stump contain stictic acid.

***Xylographavitiligo* (Ach.) J.R. Laundon**

**Basilicata** • Potenza Prov., Piana del Pollino, NW Serra delle Ciavole; 39°55'09"N, 16°12'57"E; 1900 m; 02 Jun. 1979; Mayrhofer leg.; on *Pinusleucodermis*; GZU – Ma 1180.

A mainly boreal-montane species found on decaying, decorticated but still hard wood, mostly of conifers, especially near the base, or on fallen trunks, with optimum near treeline.

## Discussion

The list includes 225 records of 153 taxa. Twenty taxa are new to Italy, the others are new to one or more administrative regions; the latter include 15 second records and 5 third records for Italy.

The administrative regions with most new records are Veneto (61 records, 50 taxa), Trentino Alto Adige (38, 32 taxa), Emilia-Romagna (22, 10 taxa), Lombardia (19, 15 taxa), and Abruzzo (14, 14 taxa). From each of the other 14 regions, 12 to 1 new records are reported.

Most records come from bark (72 records, 43 taxa), calcareous rocks (47, 34 taxa) and siliceous rocks (39, 29 taxa). Fewer come from soil, plant debris, dead wood and bryophytes.

Most records are from the alpine belt (61 records, 55 taxa), followed by the subalpine (53, 44 taxa) and montane (53, 36 taxa) belts. This is due to the fact that the research activity of most of the authors is mainly centred on the Alps (e.g. Nascimbene et al. 2017; Nimis et al. 2018a; Saiz et al. 2021).

The species listed in this paper can be subdivided into the following main groups:

1. Recently-described or -resurrected species, such as Bacidina adastra, Blastenia gennargentuae, B. monticola, B. psychrophila, Calogaya rouxii, Circinaria serenensis, Flavoplaca limonia, Fuscopannaria praetermissa, Gyalideopsis helvetica, Hypotrachyna afrorevoluta, Lecanora silvae-nigrae, Placidiopsis pseudocinerea, Protoblastenia aurata, Psoroma tenue var. boreale, Punctelia jeckeri, Pyrenodesmia erodens, Ramonia interjecta, Rhizocarpon furax, Scytinium aragonii, S. imbricatum.
2. Sterile or ephemeral species, such as Aspicilia grisea, A. mashiginensis, Bellemerea subsorediza, Gyalideopsis helvetica, Lecanora cavicola, L. epibryon var. bryopsora, L. lojkaeana, Lepra schaereri, L. teneriffensis, Leptogium byssinum, Opegrapha vermicellifera, Pertusaria flavicans, Pseudothelomma ocellatum, Rinodina pityrea, Toensbergia leucococca, Violella fucata, Varicellaria rhodocarpa, Xylographa vitiligo. Several of these species are not generally rare, but were simply overlooked, undercollected or not identified at species level in previous studies mostly because a traditional recognition approach based on macro- and microscopic characteristics is not sufficient, these taxa requiring a DNA-based identification or the definition of secondary metabolites.
3. Species belonging to taxonomically critical groups, such as Anema tumidulum, Candelariella kuusamoënsis, Diplotomma-species, Lecania cyrtellina, Lempholemma intricatum, Polysporina urceolata, Psorotichia lugubris, Staurothele sapaudica, Thelidium-species, which were probably not recognized or misidentified in previous studies.
4. Species of biogeographic interest, which in Italy (or in some regions) are near the limits of their climatic optima. Most of the nationally or regionally rare lichens belong to an oceanic-suboceanic element with tropical affinities, or to a small set of continental species with their optima in the dry steppe biome, which suggests that many rare species can persist in microrefugia, i.e. sites with microclimates that support small populations of species beyond the boundaries of the climatic limits of their main distributions. This is the case of Bacidina delicata, Fuscopannaria praetermissa, Lecanora horiza and Sticta limbata, which are at the limit of their bioclimatic ranges (suboceanic and/or Mediterranean) in a continental-alpine region such as Trentino-Alto Adige, of Fuscopannaria ignobilis, mainly Tyrrhenian, a suboceanic species which is obviously restricted to a few humid sites in Molise, of Rinodina oleae, a mainly Mediterranean species, which is at the limit of its bioclimatic range in Emilia, and of Usnea flavocardia, a subatlantic species restricted to a few sites in Tyrrhenian Italy. Another example is that of C. arbuscula and C. mitis, two arctic-alpine to boreal-montane species which are widespread and common in the Alps, but are near their southern distributional limit in the Marche region (Northern Apennines). Similar is the case of R. olivaceobrunnea, an arctic-alpine species which finds the southernmost limit of its Italian distribution in the mountains of Calabria. As already observed by Aptroot and van Herk (2007) in the Netherlands, these species are those for which climate change is most likely to modify their relative patterns of commonness/rarity.
5. Species bound to rare habitats, such as old-growth forests. This is the case of e.g. Arthonia vinosa, Calicium adspersum, Cetrelia chicitae, Chaenotheca brachypoda, C. brunneola, C. phaeocephala, Chaenothecopsis debilis, Mycobilimbia epixanthoides, Scutula circumspecta, Sphinctrina leucopoda, plus some of the species listed under the previous point. Also in this case, these species are generally rare, their rarity being mainly due to the strong contraction of their habitat.

Our results indicate that, even in historically well-explored areas it is still possible to discover several new species. Even small, previously well-studied sites may provide interesting surprises. This is the case of the small plot in the Vette Feltrine (Pre-Alps, Dolomiti Bellunesi National Park), which consists of a few square meters of rock outcrops, within a peculiar site with abundant precipitations due to humid air masses from the Adriatic Sea that originate frequent fog. The collections were carried out on a NE exposed slope where the rock outcrops contain both a calciferous (dominant) and a siliceous (flint nodules and strata) component, with an alternation of exposed and protected overhanging parts that correspond to diverse microhabitats for lichens, also providing refugia for microthermic species. Another emblematic case is represented by the Paneveggio-Pale di San Martino Natural Park, an area with a very humid climate and heterogeneous geological features, that was intensively explored by Ferdinand Arnold at the end of the 19^th^ century (Dalla Torre and Sarnthein 1902) and also in recent times in the framework of studies mainly focused on lichen ecology in forest ecosystems (e.g. Nascimbene et al. 2008; Nascimbene et al. 2009). On one hand, these examples indicate that repeated, intensive, surveys are needed to reach exhaustive knowledge even of small sites. On the other hand, they indicate that further exploration should prioritise areas with rare climatic conditions and heterogeneous rock composition, corroborating the view that high geo-diversity, even at a small spatial scale, corresponds to high lichen diversity (Spitale and Nascimbene 2013).

## Conclusion

The picture of the lichen biota of Italy now has new pixels, but its grain is still coarse. On one hand, herbaria, especially when digitized, are an irreplaceable tool for further data mining allowing the re-evaluation of old records in the light of the progress of phylogenetic hypotheses and taxonomy, and should be therefore sustained and implemented with new records (see e.g. Crisci et al. 2020). On the other hand, professional floristics should gain more consideration in the scientific community, acknowledging its fundamental role in providing and updating occurrence and distributional data, which are the basis for new biogeographic hypotheses, taxonomic and ecological research, and biodiversity conservation.
